# Oxidization enhances type I ROS generation of AIE-active zwitterionic photosensitizers for photodynamic killing of drug-resistant bacteria†

**DOI:** 10.1039/d3sc00980g

**Published:** 2023-04-17

**Authors:** Jianye Gong, Lingxiu Liu, Chunbin Li, Yumao He, Jia Yu, Ying Zhang, Lina Feng, Guoyu Jiang, Jianguo Wang, Ben Zhong Tang

**Affiliations:** a College of Chemistry and Chemical Engineering, Inner Mongolia Key Laboratory of Fine Organic Synthesis, Inner Mongolia University Hohhot 010021 P. R. China jiangguoyu@mail.ipc.ac.cn wangjg@iccas.ac.cn; b School of Science and Engineering, Shenzhen Institute of Aggregate Science and Technology, The Chinese University of Hong Kong Shenzhen Guangdong 518172 China tangbenz@cuhk.edu.cn

## Abstract

Type I photosensitizers (PSs) with an aggregation-induced emission (AIE) feature have received sustained attention for their excellent theranostic performance in the treatment of clinical diseases. However, the development of AIE-active type I PSs with strong reactive oxygen species (ROS) production capacity remains a challenge due to the lack of in-depth theoretical studies on the aggregate behavior of PSs and rational design strategies. Herein, we proposed a facile oxidization strategy to enhance the ROS generation efficiency of AIE-active type I PSs. Two AIE luminogens, MPD and its oxidized product MPD-O were synthesized. Compared with MPD, the zwitterionic MPD-O showed higher ROS generation efficiency. The introduction of electron-withdrawing oxygen atoms results in the formation of intermolecular hydrogen bonds in the molecular stacking of MPD-O, which endowed MPD-O with more tightly packed arrangement in the aggregate state. Theoretical calculations demonstrated that more accessible intersystem crossing (ISC) channels and larger spin–orbit coupling (SOC) constants provide further explanation for the superior ROS generation efficiency of MPD-O, which evidenced the effectiveness of enhancing the ROS production ability by the oxidization strategy. Moreover, DAPD-O, a cationic derivative of MPD-O, was further synthesized to improve the antibacterial activity of MPD-O, showing excellent photodynamic antibacterial performance against methicillin-resistant *S. aureus* both *in vitro* and *in vivo*. This work elucidates the mechanism of the oxidization strategy for enhancing the ROS production ability of PSs and offers a new guideline for the exploitation of AIE-active type I PSs.

## Abstract 2

An efficient type I zwitterionic photosensitizer MPD-O was constructed by an oxidization strategy, providing a new guideline for the development of AIE-active type I photosensitizers.

## Graphical abstract

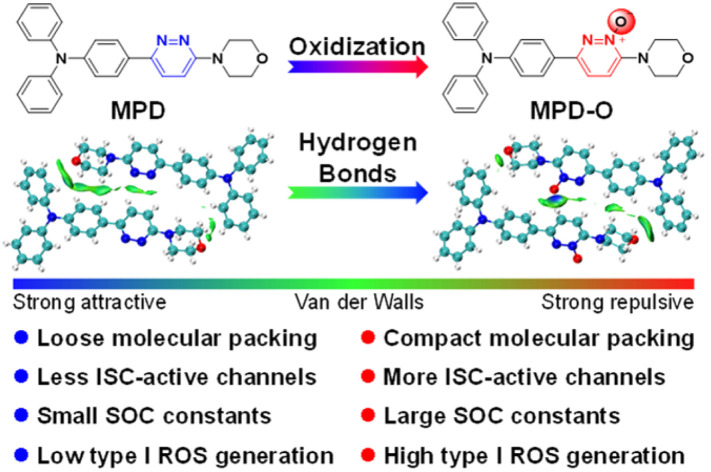

## Introduction

Photodynamic therapy (PDT), that uses external light-activated photosensitizers (PSs) to produce highly oxidizing reactive oxygen species (ROS) to induce cell or microbe death, has recently attracted considerable attention in disease therapeutics owing to its non-invasiveness, limited therapeutic resistance, excellent spatiotemporal selectivity and minimal side effects.^1–15^ According to the mechanism and type of ROS generation, there are mainly two types of PSs (type I and type II).^16–19^ To date, most of the reported PSs have come into effect mainly *via* the highly oxygen-dependent type II pathway, whereas type I PSs with low-oxygen-dependency have rarely been developed because of the lack of a universal structural design strategy, which limits the therapeutic performance of PDT to some extent.^20,21^ In addition, due to the hydrophobicity and rigid planarity, most traditional PSs easily encounter the effect of aggregation-caused quenching (ACQ) in the physiological environment, showing weak emission and poor ROS generation efficiency, consequently leading to reduced theranostic performance.^22–25^ Fortunately, aggregate science, represented by the concept of aggregation-induced emission (AIE), shows great potential to address this problem.^26–29^ AIE-active PSs usually exhibit strong emission and an aggregation-induced ROS generation effect and thus provide new opportunities for the development of PDT.^30,31^ Recently, although several AIE-active type I PSs have been developed,^32–34^ there are few reports on how to improve the ROS production efficacy of type I PSs.

Oxygen, one of the most abundant and widely distributed elements in the human body, plays significant roles in diverse vital biological and physiological processes.^35–38^ In virtue of the strong electronegativity, the electron-withdrawing properties and the capability to form diverse bonds with other atoms,^39,40^ the introduction of oxygen into PSs has the potential to significantly alter the energy level, hydrophobicity and hydrophilicity, as well as their binding capacity toward certain biological species, thus enabling effective modulation of the PS performances. Therefore, numerous methods have been developed to incorporate oxygen into molecules, among which oxidation reactions are one of the most commonly used and efficient methods.^41–43^ When electron-withdrawing oxygen atoms are introduced into PSs, we that expect the following functions to be achieved: (1) enhancement of intramolecular donor (D)–acceptor (A) interactions; (2) reduction of the energy gap between the highest occupied molecular orbital (HOMO) and lowest unoccupied molecular orbital (LUMO) of PSs; (3) promotion of bathochromic-shift of absorption and emission wavelengths; (4) strengthening of intermolecular interactions through hydrogen bonding; (5) increase of intersystem crossing (ISC) channels in the aggregated state; (6) promotion of the ROS generation capacity of PSs, and ultimately improvement of the PDT performances. Therefore, oxidation methods are a promising and effective strategy to enhance the ROS generation capacity of PSs.

**Scheme 1:**
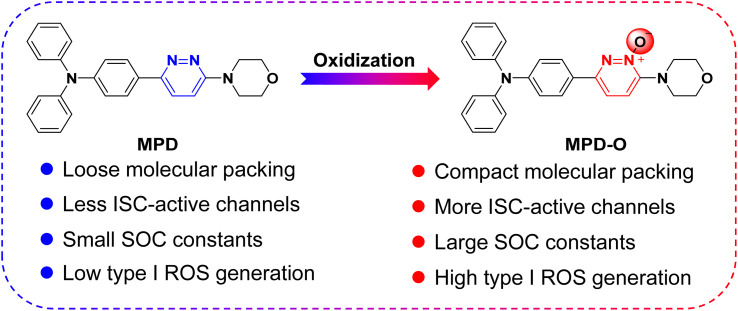
Illustration of the oxidization strategy to enhance type I ROS generation efficiency.

Zwitterions, as dipolar ions simultaneously containing positive and negative charges,^44,45^ play important roles in living organisms, taking the zwitterionic characteristics of the cell membrane as an example.^46^ Therefore, to improve the biocompatibility of PSs, zwitterionic polymers have been widely explored as an encapsulating matrix to formulate PSs into nanoparticles for biomedical applications.^47,48^ As typical zwitterions, pyridazine *N*-oxides are very important intermediates used in pharmaceutical synthesis^49–51^ and have been systematically proved to possess very low cytotoxicity and genotoxicity.^52,53^ However, as far as we know, the application of pyridazine *N*-oxides as PSs that can be achieved directly without encapsulation for disease diagnosis and treatment has not been reported. Thereafter, a facile oxidization strategy was proposed to synthesize AIE-active zwitterionic PSs with negative oxygen ions for enhancing type I ROS generation efficiency. To verify our hypothesis, two fluorophores, MPD and its oxidized product MPD-O (Scheme 1) were synthesized by two-step reactions based on 3,6-dichloropyridazine or 3,6-dichloropyridazine-1-oxide as starting raw materials. Experimental results demonstrated that both MPD and MPD-O presented typical AIE features and type I ROS generation ability. Interestingly, compared with MPD, the oxidation product MPD-O as a zwitterion exhibited higher ROS production efficiency. This is entirely consistent with our assumptions. Single-crystal analysis and independent gradient model (IGM) analysis^54,55^ revealed that MPD and MPD-O had similar molecular packing mode, but MPD-O displayed more compact molecular packing and stronger intermolecular interactions induced by more plentiful N–O⋯H interactions. Theoretical calculations demonstrated that the dimers of MPD-O with a narrower energy gap had more accessible ISC channels and larger spin–orbit coupling (SOC) constants than the dimers of MPD, which leads to higher ROS generation efficiency of MPD-O in aggregate states. To increase the binding affinity between MPD-O and bacteria, a cationic MPD-O derivative, namely DAPD-O, was synthesized, and showed an excellent and selective photodynamic effect against drug-resistant Gram-positive bacteria both *in vitro* and *in vivo*. Collectively, the work demonstrates a promising oxidization strategy to realize highly efficient type I AIE-active PSs for photodynamic killing of drug-resistant bacteria.

## Results and discussion

**Fig. 1:**
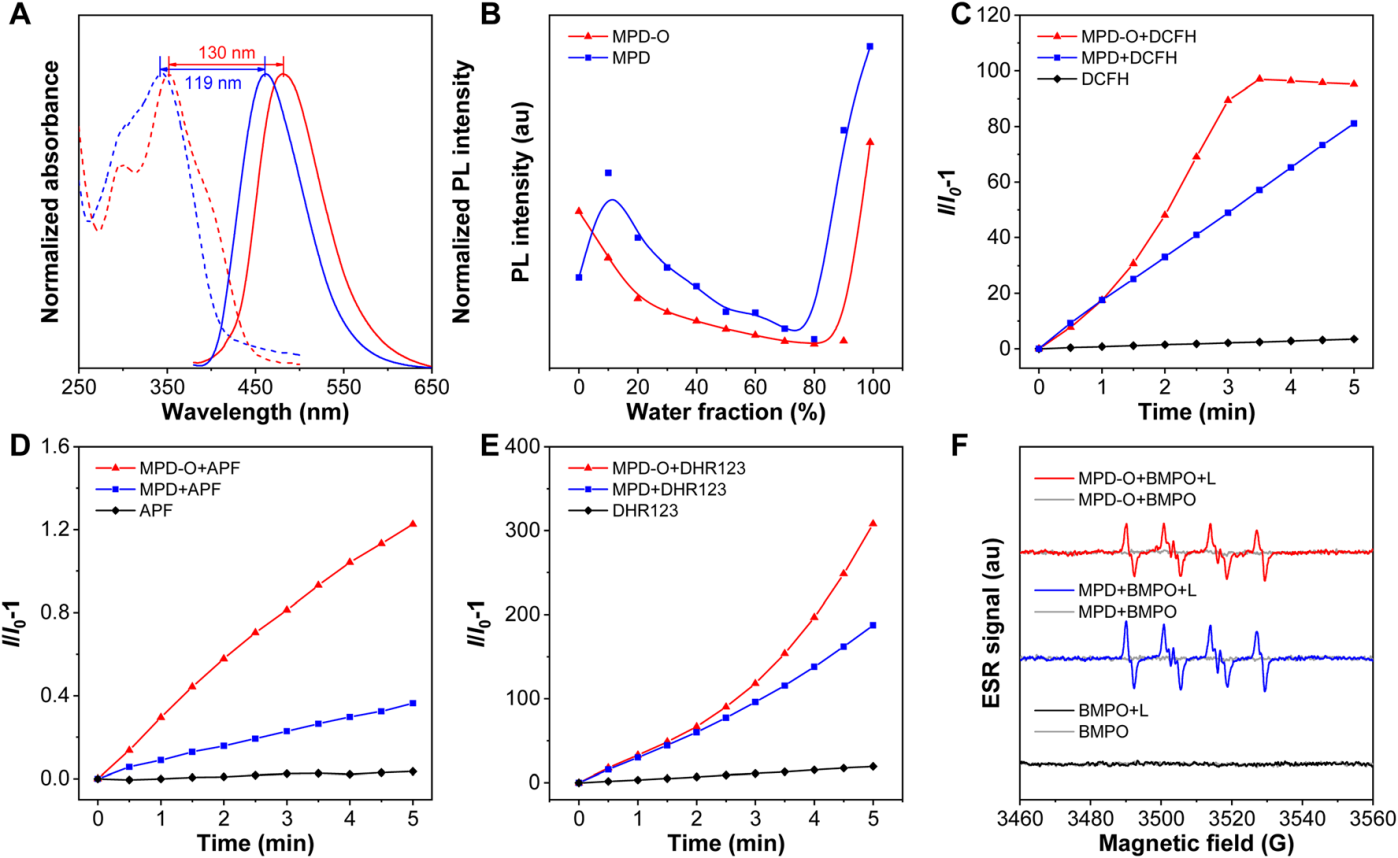
(A) Normalized absorption spectra (dashed line) and PL spectra (solid line) of MPD (blue) and MPD-O (red) in DMSO solution. (B) Plot of the PL intensity of MPD and MPD-O at the maximum emission wavelength *versus* water fraction in THF/water mixtures. ROS production of MPD and MPD-O after exposed to white light: relative changes in the PL intensity of (C) DCFH, (D) APF and (E) DHR 123 in the presence of MPD and MPD-O (10 μM) in DMSO/PBS (v/v, 1/99) upon white light irradiation (100 mW cm^−2^) for different times. (F) ESR signals of BMPO with/without MPD or MPD-O in the dark or under white light.

Firstly, compounds MPD and MPD-O were designed and synthesized through two-step reactions in high yields by employing 3,6-dichloropyridazine and 3,6-dichloropyridazine-1-oxide as starting raw materials (Schemes S1 and S2†).^56^ The structures of MPD, MPD-O, and all intermediates have been confirmed by ^1^H NMR, ^13^C NMR, and high-resolution mass spectrometry (HRMS) (Fig. S1–S11†). The photophysical properties of MPD and MPD-O were first recorded by UV-vis and photoluminescence (PL) spectroscopy. The absorption spectra of MPD and MPD-O in Fig. 1A showed a band ranging from 260 nm to 450 nm, and the absorption maxima in dimethylsulfoxide (DMSO) peaked at 342 nm and 352 nm, respectively. The maximal emission peaks were observed at 461 nm and 482 nm for MPD and MPD-O, respectively. Significantly, MPD-O showed redshifted absorption and emission spectra compared to MPD. Theoretical calculations were carried out based on density functional theory (DFT). The energy gap of the HOMO and LUMO was calculated to be 3.73 eV for MPD and 3.59 eV for MPD-O, respectively (Fig. S12†), which were consistent with the experimental results. As we expected, the introduction of electron-withdrawing oxygen atoms can enhance intramolecular D–A interactions and narrow the energy gap between the HOMO and LUMO, leading to redshifted absorption and emission wavelengths of MPD-O. The optical spectra data and theoretical calculation results preliminary confirmed our expectations. In addition, the AIE features of MPD and MPD-O were then investigated in THF/water mixed solution with varied water fractions (*f*_w_). The results in Fig. 1B and S13† showed that the PL intensity of MPD and MPD-O decreased slowly in THF/water mixtures with the water fraction lower than 80% and 90%, respectively, attributable to the twisted intramolecular charge transfer (TICT) effect. The PL intensity of MPD and MPD-O increased swiftly with further increase of the water fraction, indicating their typical AIE features. As a poor solvent for MPD and MPD-O, the addition of water would lead to the formation of aggregates, resulting in greatly restricted motion of the benzene ring in triphenylamine and thus enhancing the emission. This has then been evidenced by dynamic light scattering (DLS) analysis (Fig. S14†). The average hydrodynamic size of MPD and MPD-O was found to be 153 nm and 207 nm, respectively, in a DMSO/H_2_O (v/v, 1/99) mixture.

To estimate the photosensitizing capacities of MPD and MPD-O in the aggregate state, their overall ROS generation in DMSO/PBS (v/v, 1/99) was investigated using a ROS indicator 2′,7′-dichlorodihydrofluorescein (DCFH), which could be converted to 2′,7′-dichlorofluorescin (DCF) with green fluorescence after being activated by ROS. As depicted in Fig. 1C and S15,† the fluorescence emission of DCFH alone varied negligibly under continuous exposure to white light. However, the PL intensity of DCFH increased swiftly with MPD or MPD-O, indicating that these AIE luminogens (AIEgens) can efficiently generate ROS. Remarkably, the PL intensity of DCFH showed about 100-fold enhancement (compared to DCFH alone) with MPD-O after white light irradiation for 3 min, exhibiting obviously superior overall ROS generation efficiency than MPD (48-fold). Then different ROS indicators were utilized to confirm which kind of ROS has been generated. First, ˙OH was examined by employing aminophenyl fluorescein (APF) as an indicator, whose PL intensity will be greatly increased after reaction with ˙OH. In Fig. 1D and S16,† stark emission increment of APF has been recorded with MPD or MPD-O, especially for MPD-O, indicating their ˙OH generation capacity. Then, obvious emission enhancements were similarly captured with dihydrorhodamine 123 (DHR123) as an O_2_˙^−^ indicator (Fig. 1E and S17†). However, when 9,10-anthracenediyl-bis(methylene) dimalonic acid (ABDA) was adopted for the assessment of ^1^O_2_, no distinct signal change of ABDA was found, demonstrating poor ^1^O_2_ generation ability of MPD and MPD-O (Fig. S18†). In addition, electron spin resonance (ESR) spectroscopy was carried out with 5-*tert*-butoxycarbonyl-5-methyl-1-pyrroline-*N*-oxide (BMPO) and 4-amino-2,2,6,6-tetramethylpiperidine (TEMP) as spin-trap agents for free radicals and ^1^O_2_, respectively.^57^ In Fig. 1F and S19,† compared to the control and dark groups, MPD and MPD-O showed a typical ESR signal of free radicals with BMPO upon white light irradiation. In stark contrast, no ^1^O_2_ ESR signal of MPD or MPD-O with TEMP was observed after white light irradiation. Taken together, these results solidly evidenced that both MPD and MPD-O had type I ROS generation capacity and MPD-O indeed outperformed MPD.

**Fig. 2:**
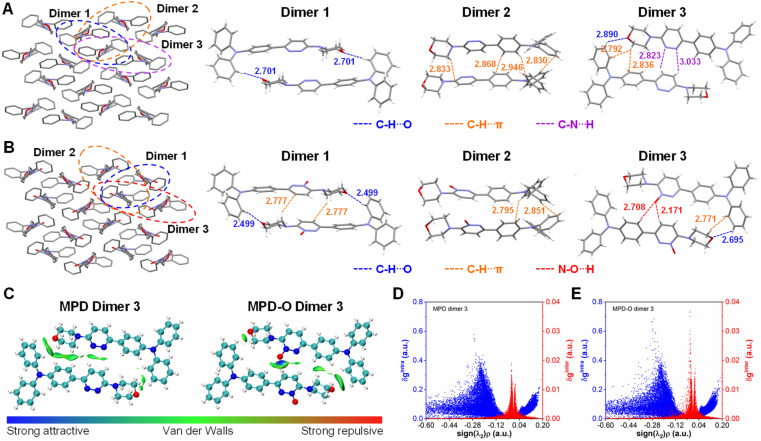
Molecular packing and intermolecular interactions of dimers in (A) MPD and (B) MPD-O single crystals. (C) The visualized isosurfaces of the IGM analysis for dimer 3 in MPD or MPD-O (*δg*^inter^ = 0.007). The 2D plot of *δg*^intra^ (blue) and *δg*^inter^ (red) for (D) MPD dimer 3 and (E) MPD-O dimer 3.

To understand why oxidization could enhance type I ROS generation efficiency, the molecular packing was investigated through single crystal analysis. The single crystals of MPD (CCDC: 2210455) and MPD-O (CCDC: 2210454) were obtained by slow evaporation of an ethyl acetate/hexane mixture (Table S1†). Two crystals belonged to the same monoclinic system with the *P*2_1_/*c* space group and exhibited highly similar molecular packing mode (Fig. 2A and B), indicating that the introduction of oxygen atoms has a negligible perturbation effect on the molecular packing. However, the presence of oxygen indeed plays some important roles in influencing the intermolecular interactions. Adjacent dimers were extracted from MPD and MPD-O crystals for detailed analysis. Notably, three similar dimers were found with different intermolecular interactions in MPD and MPD-O crystals, respectively. In MPD, only C–H⋯O interactions (2.701 Å) were observed in dimer 1, and only C–H⋯π interactions (2.830–2.946 Å) existed in dimer 2. The dimer 3 of MPD contained three types of interactions, including C–H⋯O interactions (2.890 Å), C–H⋯π interactions (2.792–2.836 Å), and C–N⋯H interactions (2.823–3.033 Å). By contrast, in MPD-O, much stronger C–H⋯O interactions with a distance of 2.499 Å in dimer 1 were recorded compared to dimer 1 of MPD. Simultaneously, new C–H⋯π interactions (2.777 Å) were also served in dimer 1 of MPD-O, implying tightly molecular packing. Intermolecular interactions were also strengthened in dimer 2 and dimer 3 of MPD-O, and both C–H⋯π interactions (2.771–2.851 Å) and C–H⋯O interactions (2.695 Å) are stronger than those in dimer 2 and dimer 3 of MPD. Particularly, thanks to the introduction of electron-withdrawing oxygen atoms, N–O⋯H interactions with distances of 2.171–2.708 Å were formed in dimer 3 of MDP-O. The formation of hydrogen bonds not only successfully enriched the types of intermolecular interactions, but also strengthened other intermolecular short contacts. To further confirm the existence of the hydrogen bond interactions, IGM analysis was conducted by using the Multiwfn program.^58^ The visual molecular dynamics (VMD) program^59^ was used to visualize the isosurfaces of weak interactions. As shown in Fig. 2C, for MPD-O dimer 3, an obvious blue region in the isosurface was observed between the electron-withdrawing oxygen atom and hydrogen atoms of the adjacent molecule, indicating the formation of strong intermolecular attraction. In contrast, there is no blue region observed in MPD dimer 3. The corresponding 2D plot of *δg*^inter^ (the descriptor for defining intermolecular interaction regions) also showed that MPD-O dimer 3 had stronger intermolecular interactions, which is in agreement with the results of intermolecular interaction distance analysis (Fig. 2D and E). We further investigated the contribution of atomic pairs to the total interaction between two adjacent molecules (Fig. S20†). The percentage contribution from atomic pairs of 19&57 and 11&57 in MPD-O dimer 3 reached 3.76% and 1.66%, respectively, which ranked first and sixth among all atomic pairs. However, the percentage contribution of 4&95 was only 1.57% for total interaction in MPD dimer 3. The IGM analysis results of other dimers are shown in Fig. S21–S24.† These results visualized the formation of hydrogen bonds and further illustrated the importance of introducing oxygen atoms. Collectively, the strengthening and promoting effect of plentiful hydrogen bonds together endowed MPD-O with a more compact packing structure in aggregate states, which ultimately leads to efficient ROS generation capacity.

**Fig. 3:**
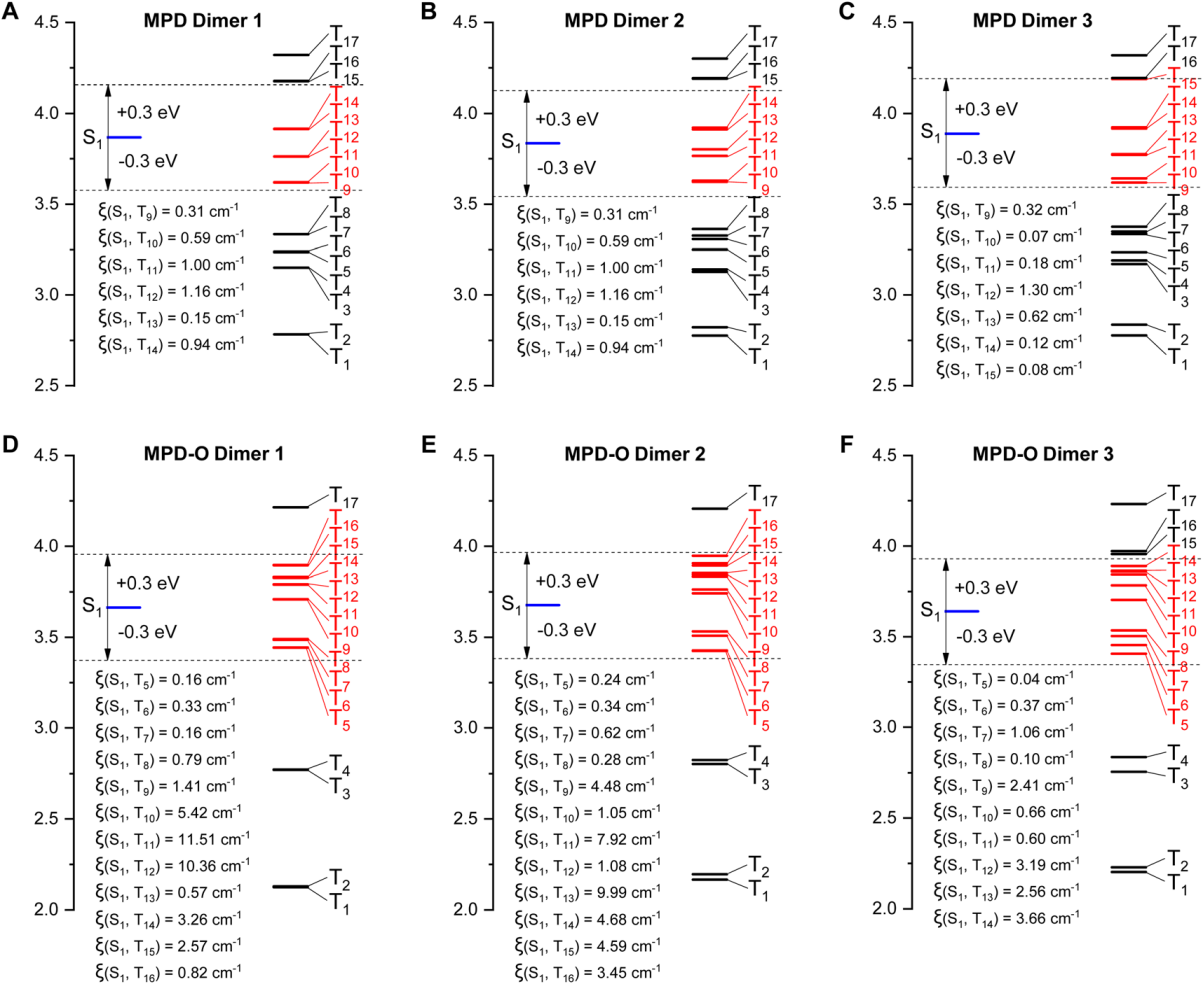
Calculated ISC channels and SOC constants (*ξ*) between S_1_ and T_n_ for the three dimers of MPD (A–C) and MPD-O (D–F) based on single crystals.

To gain an in-depth insight into how the tight packing contributed by the oxidization strategy boosts ROS efficiency, time-dependent density functional theory (TD-DFT) was conducted. It has been reported that the effective ISC channels between S_1_ and T_n_ could be realized within a maximal single-triplet energy gap with |Δ*E*_ST_| ≤ 0.3 eV.^60,61^ As shown in Fig. 3, for the three dimers of MPD, there are six, six, and seven main ISC transition channels, respectively. Remarkably, increased numbers of ISC channels (twelve, twelve, and ten channels) were obtained for MPD-O dimers. Furthermore, the related SOC constants (*ξ*) of these dimers were calculated. The maximal *ξ* of the three MPD dimers was only 1.16 cm^−1^, 1.16 cm^−1^, and 1.30 cm^−1^, respectively. However, the *ξ* values of the three MPD-O dimers, which were up to 11.51 cm^−1^, 9.99 cm^−1^, and 3.66 cm^−1^, respectively, were significantly larger than those of MPD. These results indicated that more accessible ISC channels and larger *ξ* were the reason that MPD-O had higher ROS generation efficiency in aggregate states. Generally, with the introduction of electron-withdrawing oxygen atoms, a compact packing could be formed for MPD-O in aggregate states, which could effectively facilitate the ISC process to produce triplet excitons. In other words, the oxidization strategy can effectively enhance ROS generation efficiency by regulating molecular packing. In addition, the frontier molecular orbitals of these dimers were determined (Fig. S25–S27†). Sharply different from the monomer, obvious intermolecular charge transfer characteristics were observed in MPD and MPD-O dimers, consistent with redshifted emission of aggregates in our experimental results.

**Fig. 4:**
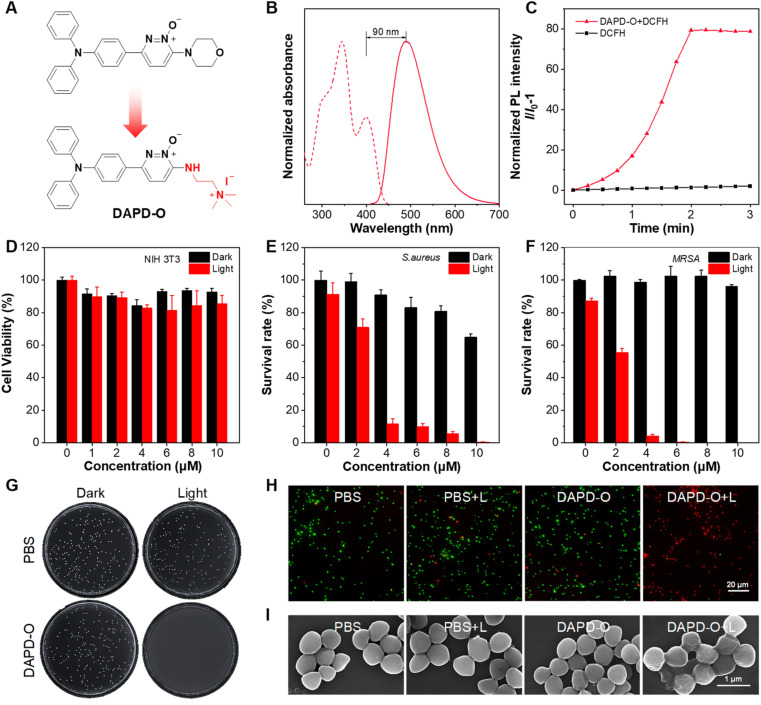
(A) Molecular structure of DAPD-O. (B) Normalized absorption spectra (dash line) and PL spectra (solid line) of DAPD-O in DMSO solution. (C) Relative changes in the PL intensity of DCFH in the presence of DAPD-O (10 μM) in DMSO/PBS (v/v, 1/99) upon white light irradiation (100 mW cm^−2^) for different times. (D) Viability of NIH-3T3 cells incubated with various concentrations of DAPD-O with/without white light (100 mW cm^−2^). Bacteria survival rates of (E) *S. aureus* and (F) MRSA exposed to DAPD-O (0–10 μM) with/without white light (100 mW cm^−2^). (G) Photographs of MRSA cultured on an agar plate after being treated with PBS or DAPD-O (10 μM). (H) Live/dead bacteria staining images of MRSA after being treated with PBS or DAPD-O (10 μM). (I) SEM images of MRSA incubated with PBS or DAPD-O (10 μM).

Diseases caused by Gram-positive bacterial infection have put human health in danger.^62–65^ Inspired by the high ROS generation efficiency of MPD-O, its photodynamic antibacterial activity was evaluated through the colony-forming units (CFU) plate count method.^66^ It was a little pity that the survival rate of bacteria remained over 30% even at a concentration of 20 μM MPD-O due to its electronic neutrality (Fig. S28†). According to reported literature,^67,68^ cationization is an effective method that can enhance the binding ability between PSs and bacteria. Therefore, a cationic MPD-O derivative, namely DAPD-O, was designed and synthesized (Fig. 4A and Scheme S3†). The chemical structures of DAPD-O were characterized by ^1^H NMR, ^13^C NMR, and HRMS (Fig. S29–S34†). Upon photoexcitation at 400 nm, the emission peak of DAPD-O was located at 490 nm, which was slightly redshifted compared to MPD-O (Fig. 4B). DLS measurements revealed that the average hydrodynamic size of DAPD-O was 160 nm in DMSO/H_2_O (v/v, 1/99) (Fig. S35†), demonstrating the formation of aggregates. As expected, the PL intensity of DCFH with DAPD-O reached 59-fold that of DCFH alone after white light irradiation for 2 min, indicating that DAPD-O also had strong ROS generation efficiency (Fig. 4C and S36†).

Before being used for antibacterial therapy, the biocompatibility of PSs must be considered. Antibacterial PSs should not damage mammalian cells when they treat bacterial infection. Therefore, the cytotoxicity of DAPD-O toward NIH-3T3 cells was assessed by cell counting kit-8 (CCK8) assay. Cell viability remained over 80% with DAPD-O (10 μM) both in darkness and under light irradiation (Fig. 4D), indicating good biocompatibility of DAPD-O. Furthermore, efficient binding of PSs toward bacteria is the prerequisite of antibacterial therapy. Thus, the fluorescence imaging performance of DAPD-O toward Gram-positive *S. aureus* and methicillin-resistant *S. aureus* (MRSA) was investigated to confirm their efficient binding toward bacteria (Fig. S37 and S38†). After being incubated with DAPD-O for 2 h, blue fluorescence signals were clearly observed inside bacteria cells, showing the efficient binding of DAPD-O toward *S. aureus* and MRSA. Inspired by the good biocompatibility, efficient binding ability and excellent ROS generation of DAPD-O, we subsequently investigated its photodynamic effect against Gram-positive *S. aureus*, MRSA, and Gram-negative *E. coli*. As shown in Fig. 4E and S39,† in the absence of DAPD-O, the survival rate of *S. aureus* diminished as DAPD-O concentration increases under white light. When the concentration of DAPD-O reached 10 μM, a survival rate over 60% was found in the dark group. While in stark contrast, *S. aureus* was killed effectively with a killing rate exceeding 99% upon light irradiation. Moreover, DAPD-O had a strong photodynamic killing effect on MRSA. Upon white light irradiation, MRSA was nearly 100% eliminated, and no obvious bacterial colony was formed on the agar plates (Fig. 4F and G). DAPD-O showed negligible dark toxicity toward MRSA, which is attributed to the special biofilm structure of drug-resistant bacteria. Meanwhile, the antibacterial effects of DAPD-O on *E. coli* were tested, and the survival rates remained at more than 80% (Fig. S40 and S41†). These results suggested that DAPD-O could selectively eliminate Gram-positive bacteria and even drug-resistant Gram-positive bacteria through PDT. Next, live/dead fluorescent staining by using calcein-AM (green channel) and PI (red channel) was conducted. As shown in Fig. S42† and 4H, the PBS group with/without white light irradiation and the DAPD-O dark group showed a large proportion of green fluorescence, indicating a high survival rate of bacteria. While after white light irradiation, almost all bacteria displayed strong red fluorescence in the presence of DAPD-O. These results were in accordance with the data from the plate count method. Meanwhile, scanning electron microscopy (SEM) was utilized to visualize the morphology changes of *S. aureus* and MRSA after PDT treatment with DAPD-O (Fig. S43† and 4I). For PBS, PBS + L, and DAPD-O groups, the cell membranes of *S. aureus* and MRSA were clear and smooth. In contrast, after PDT treatment with DAPD-O, the cell membranes of bacteria were damaged and wrinkled. These results further illustrated the photodynamic antibacterial ability of DAPD-O.

**Fig. 5:**
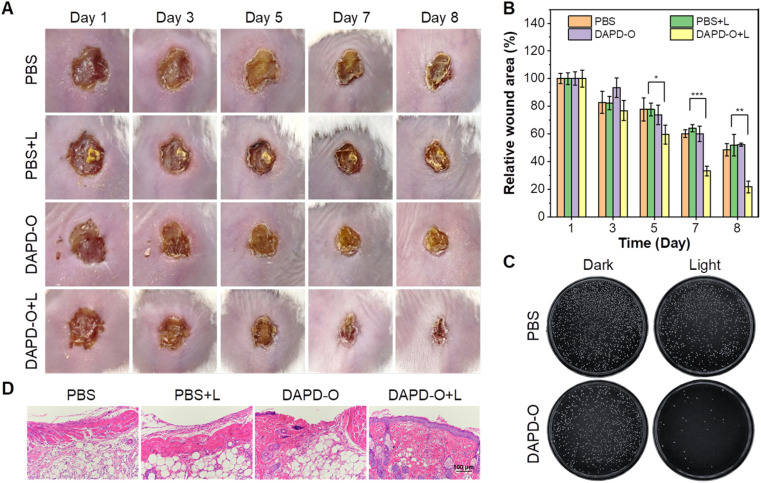
Antibacterial activity against MRSA infection *in vivo*. (A) Photographs of MRSA infected wounds treated with PBS or DAPD-O (10 μM) in darkness or upon white light irradiation (100 mW cm^−2^). (B) Relative wound area during the wound healing process after different treatments. Error bar: mean ± SD (*n* = 3, *p < 0.05, **p < 0.005, and ***p < 0.001). (C) Photographs of colony formation from wound tissues with different treatments on day 8. (D) H&E staining images of wound tissues after treatment for 8 days.

Based on the antibacterial effects of DAPD-O *in vitro*, we further evaluated its potential to promote the wound-healing process. MRSA-infected wounds on the dorsal skin of mice were prepared, and the mice were randomly divided into four groups, including PBS only (PBS group), PBS with white irradiation (PBS + L group), DAPD-O only (DAPD-O group), and DAPD-O with white irradiation (DAPD-O + L group). We take photos of the megascopic appearance of the wounds during the healing process. As shown in Fig. 5A, on day 5, the infected mice in the DAPD-O + L group exhibited a faster healing rate than those of the other groups; the sizes of the wounds were obviously smaller. On day 8, the MRSA-infected wounds in the DAPD-O + L group were largely recovered, while the wounds in the other groups still had scabs. Quantitative analysis showed that the wound area in the four groups was diminished by 51.5%, 48.2%, 47.7%, and 78.4%, respectively (Fig. 5B). To further evaluate the antibacterial effect of DAPD-O, skin tissues of the wound were extracted for bacterial culture (Fig. 5C). The DAPD-O + L group showed much fewer bacterial colonies than the other groups. This result showed that DAPD-O could effectively kill bacteria in wounds. Moreover, the wound-healing efficacy of the sectioned tissues was assessed by hematoxylin and eosin (H&E) staining. As shown in Fig. 5D, on day 8, intact epidermis and closely aligned subcuticular cells were observed in the DAPD-O + L group, while no obvious epidermis appeared in the other groups, suggesting the excellent wound-healing efficacy of DAPD-O after light treatment. What's more, the body weight of the mice remained within the normal range, displaying the good biosafety of DAPD-O (Fig. S44†). For deeply evaluating the biocompatibility of DAPD-O, blood routine assays were carried out. The hematological parameters of each group had no significant difference, and all results were in a normal range, indicating the negligible systemic toxicity of DAPD-O (Fig. S45†). These *in vivo* experimental results successfully demonstrated that DAPD-O could effectively eliminate drug-resistant Gram-positive bacteria and promote wound recovery.

## Conclusions

In summary, we proposed an oxidization strategy to boost the type I ROS generation efficiency of AIE-active PSs. As a proof-of-concept, two AIEgens, MPD and its oxidized product MPD-O were designed and synthesized. Interestingly, the zwitterion MPD-O showed higher type I ROS generation efficiency than its parent analogue MPD. Single crystal analysis and IGM analysis showed that the introduction of electron-withdrawing oxygen atoms induced the formation of intermolecular hydrogen bonds in MPD-O, leading to compact packing in the aggregate state. Theoretical calculation results further demonstrated that the compact packing promoted more accessible ISC channels and larger *ξ*, ultimately resulting in the high ROS generation efficiency of MPD-O. To further extent the antibacterial ability of MPD-O, cationic zwitterion DAPD-O was synthesized and exhibited excellent photodynamic killing Gram-positive bacteria capacity *in vitro*. Notably, DAPD-O could effectively promote the recovery of infected wounds *in vivo*. Hence, this oxidization strategy not only provides a facile method to design type I AIE-active PSs for photodynamic killing of drug-resistant bacteria, but also offers new insights into the research of zwitterionic aggregate behavior.

## Data availability

All the data supporting this article have been included in the main text and the ESI.†

## Author contributions

Guoyu Jiang, Jianguo Wang and Ben Zhong Tang contributed to the conceptualization and supervision. Jianye Gong, Lingxiu Liu, Chunbin Li, Yumao He, Jia Yu, Ying Zhang and Lina Feng contributed to the methodology. Jianye Gong, Chunbin Li contributed to the software. Jianye Gong, Guoyu Jiang and Jianguo Wang contributed to the original draft writing. Jianye Gong, Guoyu Jiang, Jianguo Wang and Ben Zhong Tang contributed to the review & editing.

## Conflicts of interest

There are no conflicts to declare.

## Supplementary Material

SC-014-D3SC00980G-s001

SC-014-D3SC00980G-s002
