# Supplementary material for: Oxidization enhances type I ROS generation of AIE-active zwitterionic photosensitizers for photodynamic killing of drug-resistant bacteria
Source: Chem Sci. 2023 Apr 17;14(18):4863–71. doi: 10.1039/d3sc00980g (PMC10171080; doi:10.1039/d3sc00980g)
Supplement: SC-014-D3SC00980G-s001 [file SC-014-D3SC00980G-s001.pdf]

Electronic Supplementary Material (ESI) for Chemical Science.
This journal is © The Royal Society of Chemistry 2023

Supporting Information

Oxidization Enhances Type I ROS Generation of AIE-Active Zwitterionic Photosensitizer for Photodynamic Killing of Drug-Resistant Bacteria

Jianye Gong,[†] Lingxiu Liu,[†] Chunbin Li,[†] Yumao He,[†] Jia Yu,[†] Ying Zhang,[†] Lina Feng,[†] Guoyu Jiang,[*,†] Jianguo Wang,[*,†] and Ben Zhong Tang[*,§]

[†]College of Chemistry and Chemical Engineering, Inner Mongolia Key Laboratory of Fine Organic Synthesis, Inner Mongolia University, Hohhot 010021, P. R. China

E-mail: jiangguoyu@mail.ipc.ac.cn; wangjg@iccas.ac.cn

[§]School of Science and Engineering, Shenzhen Institute of Aggregate Science and Technology, The Chinese University of Hong Kong, Shenzhen 518172, P. R. China

E-mail: tangbenz@cuhk.edu.cn

Experimental Procedures

Materials

N,N-diphenyl-4-(4,4,5,5-tetramethyl-1,3,2-dioxaborolan-2-yl)aniline were purchased from Soochiral chemistry. 3,6-Dichloropyridazine and morpholine were purchased from Innochem. All other chemicals and reagents were purchased from Admas-beta® and used directly without further purification. Phosphate buffered solution (PBS, pH 7.4), 2',7'-dichlorodihydrofluorescein diacetate (DCFH-DA), aminophenyl fluorescein (APF), dihydrorhodamine 123 (DHR123), 9,10-anthracenediyl-bis(methylene) dimalonic acid (ABDA), 5-tert-butoxycarbonyl-5-methyl-1-pyrroline-N-oxide (BMPO), 4-amino-2,2,6,6-tetramethylpiperidine (TEMP), calcein acetoxymethyl ester (Calcein-AM), propidium iodide (PI) were purchased from Sigma-Aldrich. Cell Counting Kit-8 (CCK-8) were purchased from Dojindo Laboratories. The *S. aureus* (BNCC 186335) and *MRSA* (BNCC 337371) were purchased from BeNa Culture Collection. The *E.coli* was obtained from the Engineering Research Center of Dairy Quality and Safety Control Technology Ministry of Education of Inner Mongolia University.

Instruments

^{1}H and ^{13}C NMR spectra were recorded with a Bruker ARX 500 NMR spectrometer using tetramethylsilane (TMS) as a reference. High resolution mass spectra (HRMS) were measured with a LCMS9030 spectrometer. UV-vis absorption spectra were recorded on a SHIMADZU UV-2600i spectrophotometer. Photoluminescence (PL) spectra were recorded on a HITACHI F-4700 fluorescence spectrophotometer. The absolute fluorescence quantum yield was measured using a Hamamatsu quantum yield spectrometer C11347-11 Quantaurus QY. Single crystal X-ray diffraction was performed on a Rigaku Oxford Diffraction Supernova Dual Source, Cu at Zero equipped with an AtlasS2 CCD using Cu Kα radiation. The data were collected and processed using CrysAlisPro. Size distribution and zeta potential were analyzed on a dynamic light scattering (DLS) using an Omni NanoBrook. The cell viability was detected by CCK-8 kit, and the absorbance of each sample was measured at 450 nm using a microplate reader (BioTek). The bacterial fluorescence images were taken by inverted fluorescence

microscope (Nikon Ti2). The bacterial morphology was observed on a HITACHI S-4800 scanning electron microscope. The photographs of agar plate were taken by an automated colony counter (Shineso Icount33).

Density functional theory calculations

The density functional theory (DFT) method was used to optimize the molecular geometries of MPD and MPD-O in gaseous state at the level of B3LYP/6-31G(d). The frontier molecular orbitals of the dimers in crystal were calculated at the level of B3LYP/6-31G(d). The excited energies were calculated by the time-dependent density functional theory (TD-DFT) method at the level of wB97XD/def2TZVP. All the calculations were performed within Gaussian 09 software package. The SOCs between singlet and triplet states were given by PySOC[1] and atomic integrals in PySOC were calculated from MolSOC code developed by Sandro Giuseppe Chiodo et al.[2] IGM analysis[3,4] of weak interaction based on single crystal structure was conducted by using Multiwfn.[5] The corresponding structure and IGM isosurfaces were generated using VMD.[6]

Total ROS Detection by DCFH

A commonly used ROS indicator 2', 7'-dichlorodihydrofluorescein diacetate (DCFH-DA) was utilized to detect the ROS generation of MPD, MPD-O and DAPD-O in aqueous solution under white light irradiation (100 mW cm^{-2}). Briefly, 0.5 mL DCFH-DA in ethanol (1×10^{-3} M) was added to 2 mL NaOH (1×10^{-2} M) and allowed to be stirred at room temperature for 30 min. Then the hydrolysate was neutralized with 10 mL of 1 × PBS at pH 7.4, and kept in dark before use. By the time, DCFH-DA was hydrolyzed to DCFH. Then the ROS indicator (4×10^{-5} M) in PBS was further diluted to 1×10^{-6} M in the sample solution of MPD, MPD-O or DAPD-O (1×10^{-5} M) for measurement by PL instrument. The fluorescence of 2', 7'-dichlorofluorescein triggered by PS sensitized ROS under white light irradiation was recorded at different time intervals. The PL spectra were measured with excitation at 489 nm and emission was collected from 500 to 600 nm. The fluorescence intensity at 525 nm was recorded to indicate the generation rate of ROS.

Detection of $O_2^{\bullet-}$ Generation by DHR123

The $O_2^{\bullet-}$ generation measurements were performed using dihydrorhodamine 123 (DHR123) as an indicator. The stock solution of DHR 123 (1 mM) was diluted to 5 μM in the sample solution of MPD or MPD-O (1×10^{-5} M) in PBS. The fluorescence signal of DHR 123 was monitored at different time intervals in a range of 500-600 nm with the excitation wavelength at 480 nm after the solution was irradiated by white light irradiation (100 mW cm^{-2}). The fluorescence intensity at 527 nm was recorded to indicate the generation rate of $O_2^{\bullet-}$.

Detection of •OH Generation by APF

The •OH generation measurements were performed using aminophenyl fluorescein (APF) as an indicator. The stock solution of APF (5 mM) was diluted to 5 μM in the sample solution of MPD or MPD-O (1×10^{-5} M) in PBS. The fluorescence signal of APF was monitored at different time intervals in a range of 500-600 nm with the excitation wavelength at 490 nm after the solution was irradiated by white light irradiation (100 mW cm^{-2}). The fluorescence intensity at 515 nm was recorded to indicate the generation rate of •OH.

Detection of $^1O_2$ Generation by ABDA

For $^1O_2$ detection indicated by 9,10-anthracenediyl-bis(methylene)-dimalonic acid (ABDA), the stock solution of ABDA (5 mM) was diluted to 40 μM in the sample solution of MPD or MPD-O (1×10^{-5} M) in PBS. The absorption spectra of ABDA were monitored in a range of 325-425 nm after the solution was irradiated by white light irradiation (100 mW cm^{-2}). The absorbance decrease of ABDA at 380 nm was recorded to indicate the decomposition rates of ABDA.

ESR Analysis

ESR measurement was used to identify the type of ROS using 5-tert-butoxycarbonyl-5-methyl-1-pyrroline-N-oxide (BMPO) as the radical indicator and 2,2,6,6-tetramethylpiperidine (TEMP) as the $^1O_2$ indicator. Samples were prepared by mixing 200 μL of MPD (20 μM) or MPD-O (20 μM) in water and 200 μL of BMPO (100 mM) or TEMP (25 mM) in water. ESR signals were

recorded by adding samples through a capillary tube under a white light irradiation at 100 mW cm^{-2} for 5 minutes.

Bacteria Culture

A single colony of *S. aureus*, *MRSA* or *E. coli* on LB agar was transferred to 10 mL of LB liquid culture medium and grown for 10 h at 37 °C with a shaking speed of 220 rpm. Bacteria were harvested by centrifuging at 4000 rpm for 7 min and washed twice with PBS (pH = 7.4). After removal of the supernatant, the remaining bacteria were resuspended in PBS, and diluted to an optical density of 1.0 at 600 nm (OD_{600} = 1.0 with about 10^9 CFU mL^{-1}).

Bacteria Staining and Imaging

After harvested by centrifugation, 1 mL of *S. aureus* or *MRSA* solution in PBS with a density of 2×10^8 CFU mL^{-1} were mixed with DAPD-O (20 μM), respectively. After dispersion with vortex, the samples were incubated at 37 °C with a shaking speed of 220 rpm for 2 h, respectively. To capture fluorescence images, 2 μL of stained bacteria solution was transferred to a piece of glass slide and then covered by a coverslip. The images were collected using an inverted fluorescence microscope. Capture conditions: PSs: λ_{ex} = 352-402 nm and λ_{em} = 417-477 nm.

Antimicrobial Assay

Bacteria (*S. aureus*) at a density of ~10^7 CFU mL^{-1} were dispersed in the solutions containing MPD-O (0, 2, 5, 10 and 20 μM). Bacteria (*S. aureus*, *E. coli* or *MRSA*) at a density of ~10^7 CFU mL^{-1} were dispersed in the solutions containing DAPD-O (0, 2, 4, 6, 8 and 10 μM). These mixed solutions were then incubated at 37°C with a shaking speed of 220 rpm for 30 min. Next, the bacterial suspensions were exposed to white light irradiation for 40 min (100 mW cm^{-2}) for phototoxicity test or were further incubated in the darkness at 37°C for assessing the dark toxicity. Afterward, the samples were diluted to a density of ~10^2 CFU mL^{-1} with 1×PBS and spread on the LB agar plate, followed by culturing at 37 °C for 16 h before colony forming units (CFU) counting and taking photos.

Cell Culture

NIH 3T3 cells were cultured in DMEM (containing 10% heat-inactivated FBS, 100 mg·mL^{-1} penicillin and 100 mg·mL^{-1} streptomycin) at 37 °C in a humidified incubator with 5% CO_2. Before the experiments, the cells were precultured until confluence was reached.

Cell viability via CCK-8 Assay

100 μL of cell suspension (5000 cells/well) were uniformly distributed in a 96-well plate. The cells were pre-incubated for 24 h at 37 ℃ in a humidified incubator. Remove old media and then add 100 μL fresh medium containing various concentrations of DAPD-O (0 μM, 1 μM, 2 μM, 4 μM, 6 μM, 8 μM, 10 μM) to the plate. After co-incubating the plate for 30 min in a humidified incubator, the plate was exposed to white light (100 mW cm^{-2}) for 40 minutes or in the darkness. The plate was normally nurtured for another 1 hours at 37 ℃. Finally, the following steps for CCK-8 test were carried out at a wavelength of 450 nm by a microplate reader. The results were expressed as the viable percentage of cells after different treatments relative to the control cells without any treatment. The relative cell viability was calculated according to the following formula: Cell viability (%) = (OD_{sample} - $OD_{background}$)/($OD_{control}$ - $OD_{background}$) × 100%.

Live/dead staining assay

Followed by antimicrobial experiments, the bacteria were collected after irradiation and incubated with Calcein-AM (10 μM) and PI (10 μM) for 1 h. Then, the bacteria were washed one time with sterile PBS. The resulting bacterial suspension (2 μL) was added onto a glass slide, which was immobilized by a clean coverslip for characterization by inverted fluorescence microscope.

SEM analysis

Followed by antimicrobial experiments, the bacteria were collected after irradiation and fixed with 2.5% glutaraldehyde overnight. The glutaraldehyde was removed by centrifugation and the bacteria were washed with PBS for 2 times. Then the bacteria were dehydrated with a series

of graded ethanol/water solution ($v_{ethanol}$ /v_{water} =10%, 30%, 50%, 70%, 80% 90%, 100%) for 15 min each. 2 μL of bacterial suspensions were added onto clean silicon slices followed by naturally drying in the air. The specimens were coated with Au before SEM analysis.

In *vivo* assay against *MRSA*

BALB/c mice (6−8 weeks old, average body weight 16−18 g) were purchased from SPF Biotechnology Co., Ltd. (Beijing, China) and all animals received care in compliance with the guidelines outlined in the Guide for the Care and Use of Laboratory Animals. All procedures were approved by the Institutional Animal Care and Use Committee at the Inner Mongolia University (IMU-2022-mouse-047). The mice were randomly divided into four groups: (1) *MRSA*-infected group with PBS in darkness; (2) *MRSA*-infected group with PBS plus white light irradiation treatment; (3) *MRSA*-infected group with DAPD-O in darkness; (4) *MRSA*-infected group with DAPD-O plus white light irradiation treatment. The mice were anesthetized by injection of 1% pentobarbital sodium saline solution (5 $mL{\cdot}kg^{-1}$), and hair removal cream was used to remove the hair on their backs for subsequent experiments. Next, a full-thickness skin wound with a diameter of 8 mm was made on the back of each mouse. Bacterial suspension (100 μL, $1{\times}10^{8}$ CFU mL^{-1}) was dripped on the surface of wounds, and the bacterial suspension were kept in the wounds for 30 minutes. Thirty minutes later, 50 μL of PBS or DAPD-O (10 μM) was sprayed on infected wounds for another 30 min, and treated with or without white light irradiation (100 mW cm^{-2}) for 40 min. In sterile environment, mice were fed separately in different cages to facilitate wound healing after operation. The wound sizes were imaged by a video camera and calculated at designated time intervals.

Histological Analysis

The wounds were histologically analyzed at day 8 post operation. Wound tissues were collected and fixed in 4% formaldehyde solution for 30 min. The pathological sections of wound tissues were analyzed by H&E staining. Histological images were taken by an inverted microscopy.

Biosafety Assessment

To further evaluate the safety of different treatments in vivo, blood samples were collected from mice with various treatments for complete blood panel analysis. White blood cell (WBC) counts, lymphocyte counts (LYMPH#), neutrophil counts (NEUT#), red blood cell (RBC), hemoglobin (HGB), and platelets (PLT) were measured.

Statistical Analysis

All data were expressed in this article as mean result ± standard deviation (s.d.). Statistically significant difference was evaluated by t-test, and statistical significance was considered as *p < 0.05, **p < 0.005 and ***p < 0.001 (n = 3).

Synthesis and Characterization

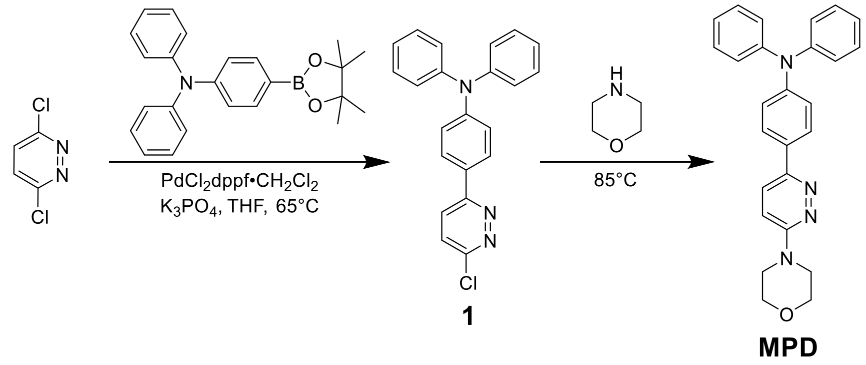

Scheme S1. Synthetic routes to MPD.

Synthesis of **1**: To a 100 mL two-neck flask equipped was added 3,6-dichloropyridazine (447 mg, 3 mmol), N,N-diphenyl-4-(4,4,5,5-tetramethyl-1,3,2-dioxaborolan-2-yl)aniline (1250 mg, 3.3 mmol) and $PdCl_2dppf•CH_2Cl_2$ (123 mg, 5 mol %). The solids were placed under an atmosphere of N_2. Freshly distilled THF (10 mL) and 2.0 M aq. K_3PO_4 (3 eq) was added via syringe. The reaction mixture was refluxed for 16 h. Then, the reaction was cooled to room temperature. The resulting slurry was suspended in CH_2Cl_2 (75 mL) and washed with H_2O (3×75 mL) followed by saturated aq. NH_4Cl (3×25 mL). The organic layer was collected, dried over Na_2SO_4, filtered, and concentrated under reduced pressure. The crude product was purified by silica gel column chromatography using petroleumether/ethyl acetate (*v*:*v*, 10:1) as an eluent to afford a beige solid of **1** (790 mg, 74% yield). ^1H NMR (500 MHz, $CDCl_3$) δ 7.93 (d, J = 8.8

Hz, 2H), 7.77 (d, J = 9.0 Hz, 1H), 7.52 (d, J = 9.0 Hz, 1H), 7.32 (t, J = 8.4 Hz, 4H), 7.19 – 7.15 (m, 6H), 7.12 (t, J = 7.4 Hz, 2H); ^{13}C NMR (126 MHz, $CDCl_3$) δ 158.10, 154.70, 150.17, 147.00, 129.51, 128.28, 127.91, 127.72, 125.38, 125.35, 123.97, 122.15.

Synthesis of **MPD**: To a 25 mL round-bottom flask was added **1** (357 mg, 1 mmol) and morpholine (1045 mg, 12 mmol). The reaction mixture was refluxed for 8 h. Then, the reaction was cooled to room temperature. The resulting solution was diluted with $CHCl_3$ (40 mL) and washed with saturated aq. $NaHCO_3$ (50 mL). The aqueous layer was then extracted with $CHCl_3$ (2×30 mL). The organic layer was collected, dried over Na_2SO_4, filtered, and concentrated under reduced pressure. The crude product was purified by silica gel column chromatography using petroleumether/ethyl acetate (*v*:*v*, 5:1) as an eluent to afford the beige solid of MPD (330 mg, 81% yield). ^{1}H NMR (500 MHz, $CDCl_3$) δ 7.90 (d, J = 8.8 Hz, 2H), 7.65 (d, J = 9.5 Hz, 1H), 7.30 (t, J = 8.5 Hz, 4H), 7.18 – 7.16 (m, 6H), 7.08 (t, J = 7.3 Hz, 2H), 6.97 (d, J = 9.6 Hz, 1H), 3.90 (t, J = 4.7 Hz, 4H), 3.69 (t, J = 5.1 Hz, 4H); ^{13}C NMR (126 MHz, $CDCl_3$) δ 158.77, 151.38, 148.63, 147.43, 130.15, 129.37, 126.77, 124.83, 124.79, 123.33, 123.13, 113.02, 66.60, 45.48; HRMS (ESI): *m/z:* $[M+H]^+$ calcd for $[C_{26}H_{25}N_4O]^+$: 409.20284; found: 409.20229.

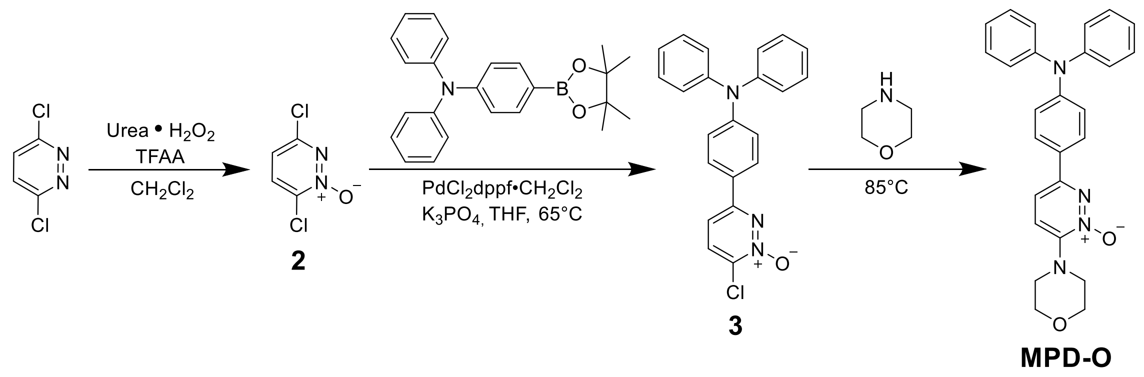

Scheme S2. Synthetic routes to MPD-O.

Synthesis of **2**: To a 250 mL two-neck flask equipped with a reflux condenser and liquid addition funnel was added 3,6-dichloropyridazine (3 g, 20 mmol), urea•H_2O_2 (7.6 g, 80 mmol) and CH_2Cl_2 (60 mL). The resulting suspension was stirred at room temperature for 15 min during which time most of the urea•H_2O_2 solubilized. Then, TFAA (11 mL, 80 mmol) was added drop wise via addition funnel over 40 min. During the course of TFAA addition, the reaction became homogeneous and warmed to 50 °C. Upon complete addition of TFAA, the reaction

was allowed to cool to ambient temperature over 1 h. The resulting suspension was further cooled to 0 °C and quenched by drop wise addition of saturated aq. $Na_2S_2O_3$ (4 mL) over 1 h. The reaction was acidified by addition of 0.5 M aq. HCl (4 mL) and extracted with CH_2Cl_2. The combined organics were washed with saturated aq. $NaHCO_3$, dried over Na_2SO_4 and filtered. The resulting solution was concentrated under reduced pressure to provide a yellow residue. Recrystallization from toluene (0.5 mL/mmol) afforded the white solid of **2** (2.6 g, 65% yield). ^{1}H NMR (500 MHz, $CDCl_3$), *δ* (ppm): 7.78 (d, *J* = 8.6 Hz, 1H), 7.13 (d, *J* = 8.6 Hz, 1H).

Synthesis of **3**: The synthetic process was similar to **1** except for the change of starting materials. Pure **3** was isolated as yellow solid with the yield of 67%. ^{1}H NMR (500 MHz, $CDCl_3$) δ 7.83 (d, *J* = 9.0 Hz, 2H), 7.77 (d, *J* = 8.6 Hz, 1H), 7.36 (d, *J* = 8.7 Hz, 1H), 7.35 – 7.32 (m, 4H), 7.18 – 7.13 (m, 6H), 7.10 (d, *J* = 8.9 Hz, 2H); ^{13}C NMR (126 MHz, $CDCl_3$) δ 157.39, 150.76, 146.68, 135.19, 134.40, 129.59, 128.11, 125.64, 125.14, 124.35, 121.31, 113.61.

Synthesis of **MPD-O**: The synthetic process was similar to MPD except for the change of starting materials. Pure MPD-O was isolated as yellow solid with the yield of 80%. ^{1}H NMR (500 MHz, $CDCl_3$), *δ* (ppm): 7.81 (d, *J* = 8.9 Hz, 2H), 7.43 (d, *J* = 8.7 Hz, 1H), 7.31 (t, *J* = 8.4 Hz, 4H), 7.22 (d, *J* = 8.7 Hz, 1H), 7.16 (d, *J* = 8.4 Hz, 4H), 7.12 – 7.09 (m, 4H), 3.97 (t, *J* = 4.6 Hz, 4H), 3.42 (t, *J* = 4.4 Hz, 4H); ^{13}C NMR (126 MHz, $CDCl_3$) δ 151.88, 149.66, 148.02, 147.05, 129.48, 127.55, 126.90, 125.22, 123.83, 122.14, 122.09, 114.80, 66.51, 47.80; HRMS (ESI): *m/z:* $[M+H]^+$ calcd for $[C_{26}H_{25}N_4O_2]^+$: 425.19775; found: 425.19720.

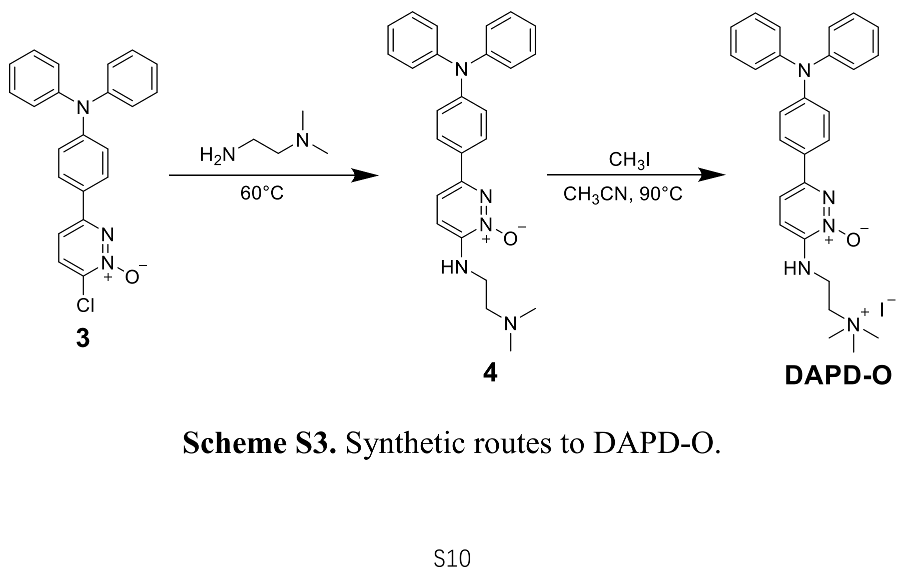

Scheme S3. Synthetic routes to DAPD-O.

Synthesis of **4**: The synthetic process was similar to **MPD-O** except for the change of starting materials. Pure **4** was isolated as yellow solid with the yield of 75%. ^{1}H NMR (500 MHz, $CDCl_3$) δ 7.78 (d, *J* = 8.8 Hz, 2H), 7.43 (d, *J* = 8.8 Hz, 1H), 7.30 (d, *J* = 8.1 Hz, 3H), 7.15 – 7.06 (m, 9H), 7.01 (d, *J* = 8.9 Hz, 1H), 6.85 (t, *J* = 5.0 Hz, 1H), 3.38 (q, *J* = 5.4 Hz, 2H), 2.68 (t, *J* = 5.4 Hz, 2H), 2.34 (s, 6H); ^{13}C NMR (126 MHz, $CDCl_3$) δ 148.92, 147.28, 146.16, 144.74, 129.38, 128.10, 127.00, 124.96, 123.50, 122.70, 116.37, 113.02, 57.55, 45.28, 39.95; HRMS (ESI): *m/z:* $[M+H]^+$ calcd for $[C_{26}H_{28}N_5O]^+$: 426.22939; found: 426.23018.

Synthesis of **DAPD-O**: To a 50 mL pressure vial was added **4** (108 mg, 0.25 mmol), iodomethane (142 mg, 1 mmol) and acetonitrile (5 mL). The resulting solution was stirred at 90 °C overnight. Then, the reaction was cooled to room temperature. The solvent was evaporated under reduced pressure. The crude solid was washed with ethyl acetate. Pure DAPD-O was obtained as yellow solid with the yield of 78%. ^{1}H NMR (500 MHz, CD_3OD) δ 7.85 (t, *J* = 9.3 Hz, 3H), 7.57 (d, *J* = 8.9 Hz, 1H), 7.33 (t, *J* = 7.8 Hz, 4H), 7.12 (d, *J* = 8.3 Hz, 6H), 7.07 (d, *J* = 8.7 Hz, 2H), 3.95 (t, *J* = 5.8 Hz, 2H), 3.68 (t, *J* = 6.5 Hz, 2H), 3.28 (s, 9H); ^{13}C NMR (126 MHz, CD_3OD) δ 149.57, 147.48, 147.16, 144.05, 129.20, 126.74, 124.97, 124.89, 123.67, 121.79, 119.00, 115.68, 63.92, 52.83, 35.85; HRMS (ESI): *m/z:* $[M-I]^+$ calcd for $[C_{27}H_{30}N_5O]^+$: 440.24449; found: 440.24315.

Supplementary Figures

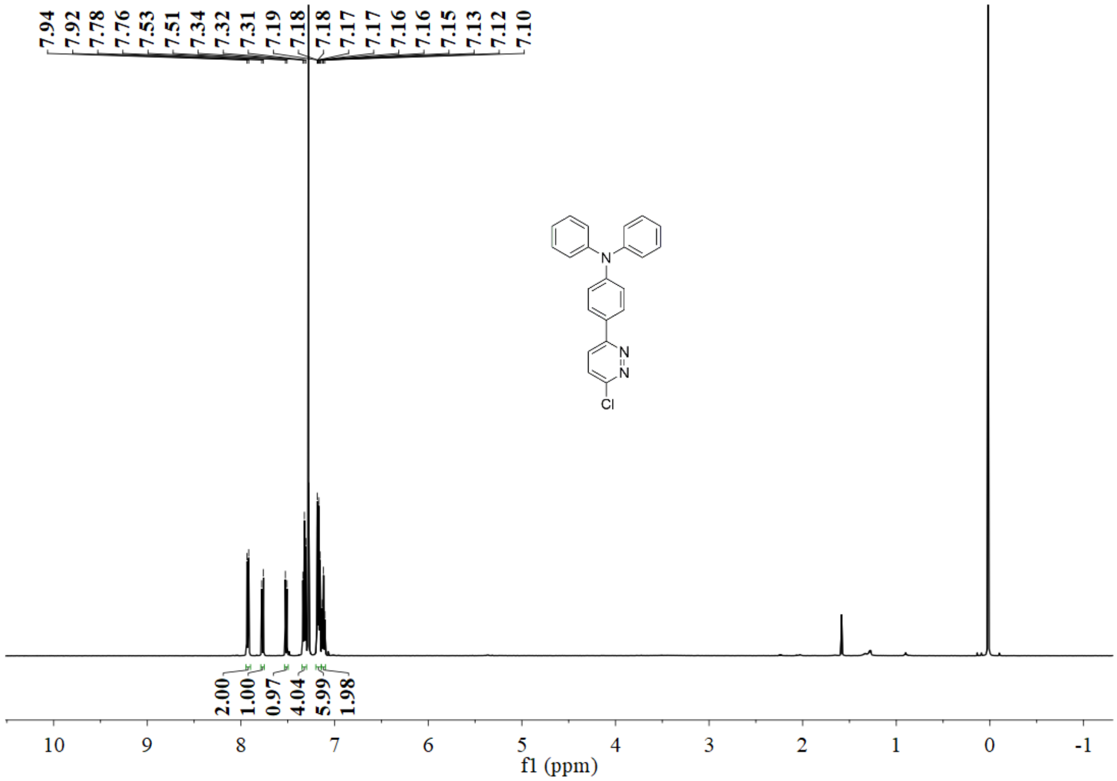

Figure S1. ^{1}H NMR spectrum of **1** in $CDCl_3$.

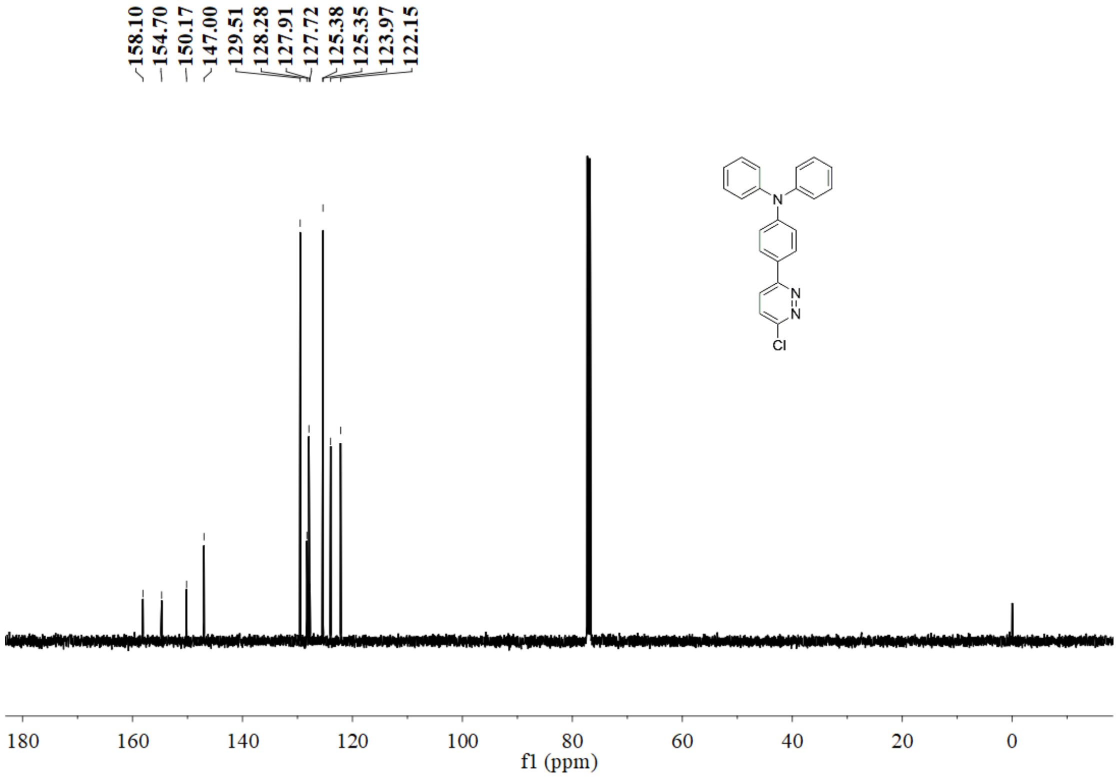

Figure S2. ^{13}C NMR spectrum of **1** in $CDCl_3$.

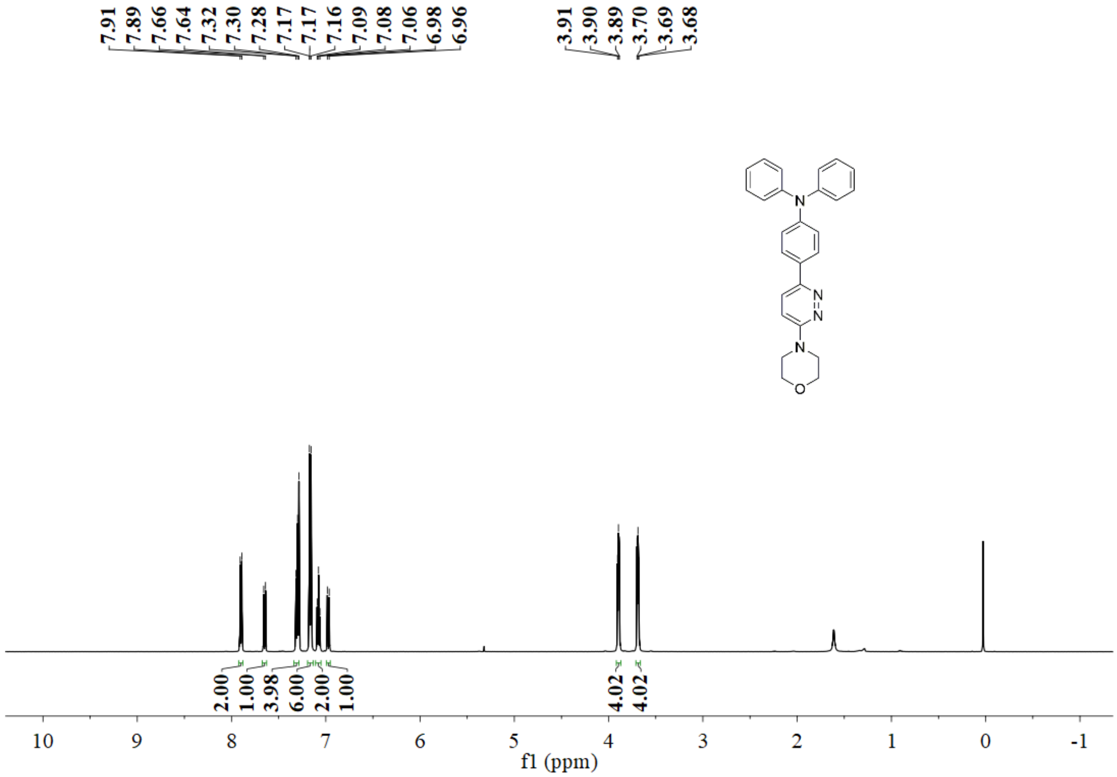

Figure S3. ^{1}H NMR spectrum of MPD in $CDCl_3$.

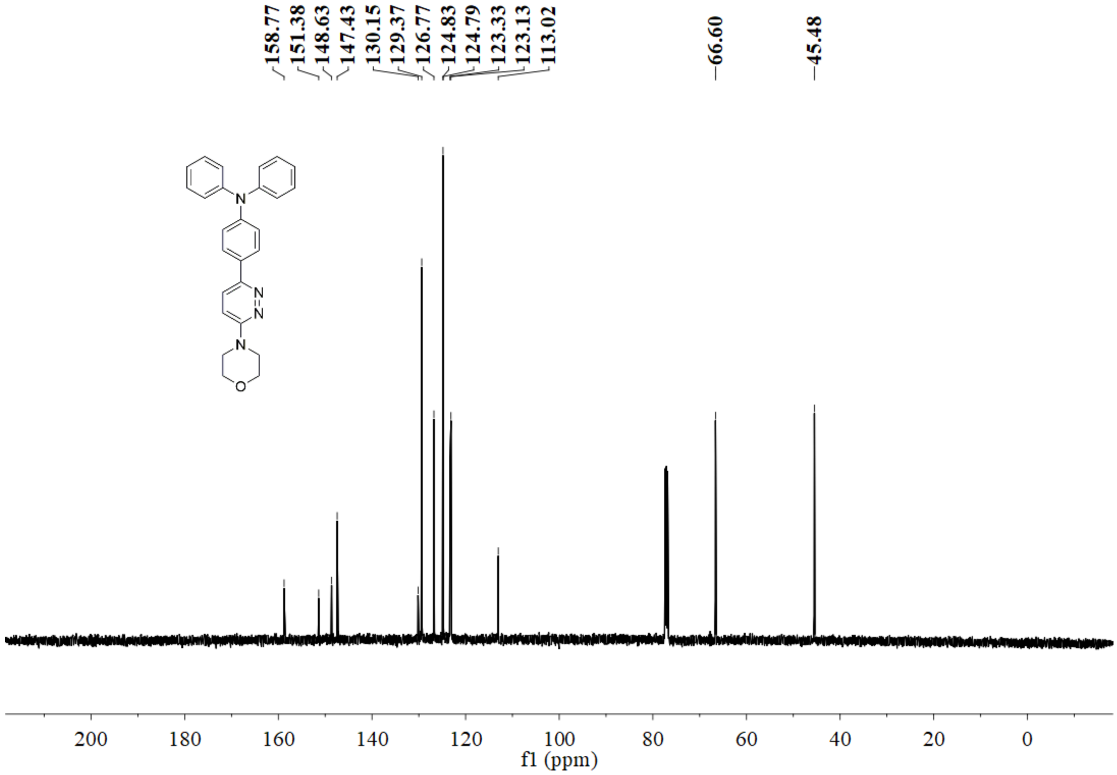

Figure S4. ^{13}C NMR spectrum of MPD in $CDCl_3$.

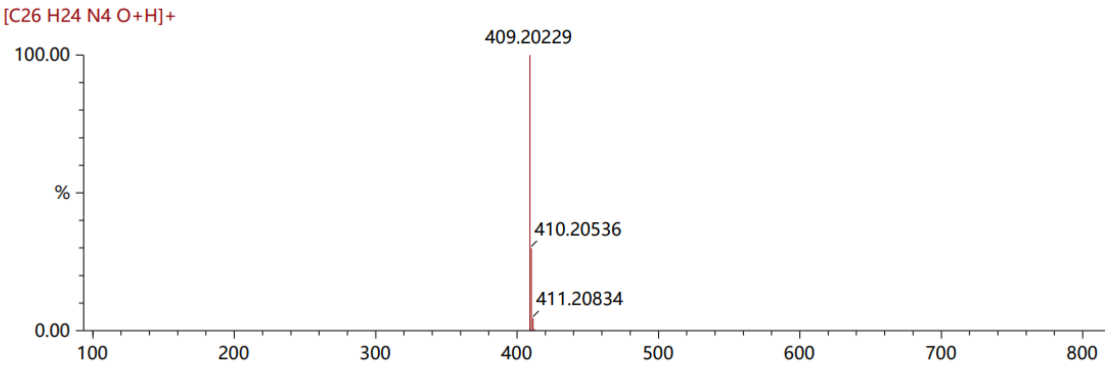

Figure S5. HRMS spectrum of MPD.

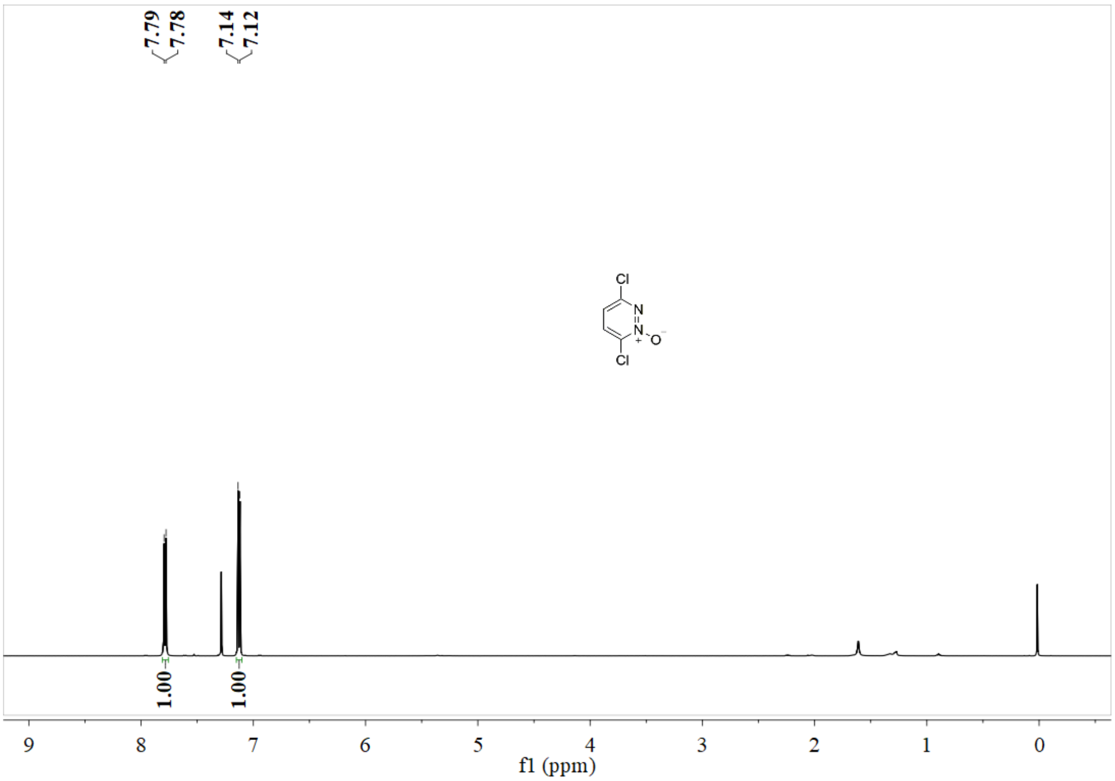

Figure S6. ^{1}H NMR spectrum of **2** in $CDCl_3$.

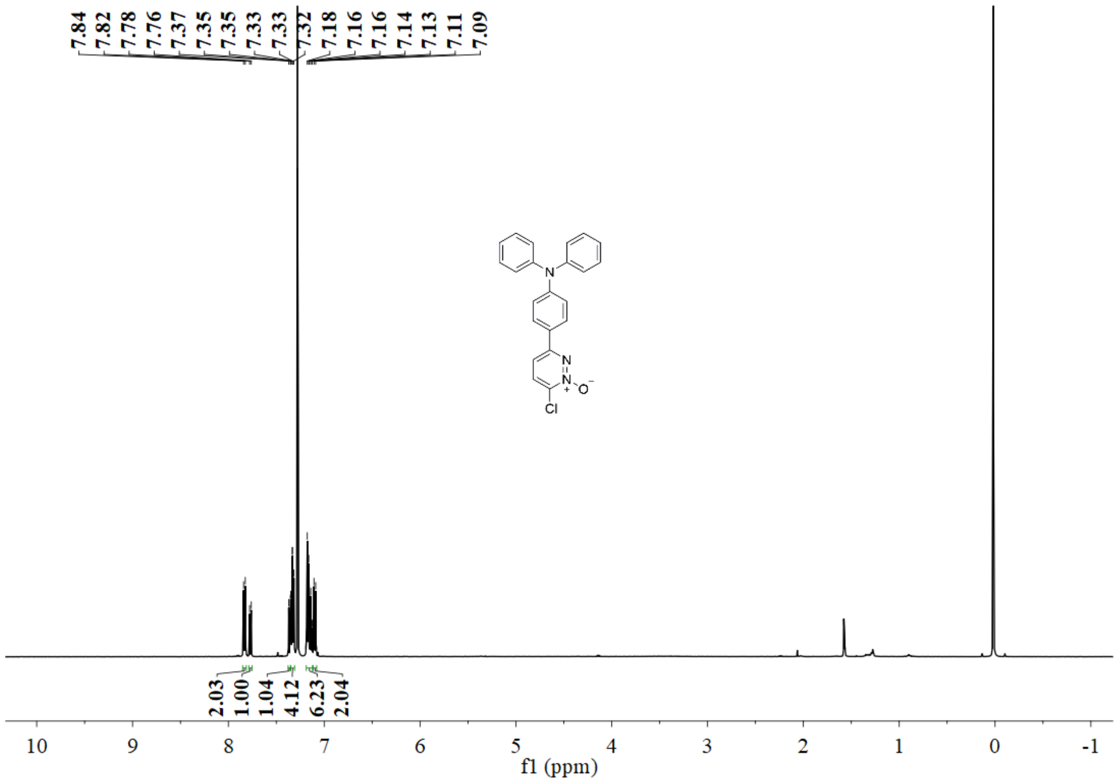

Figure S7. ^{1}H NMR spectrum of **3** in $CDCl_3$.

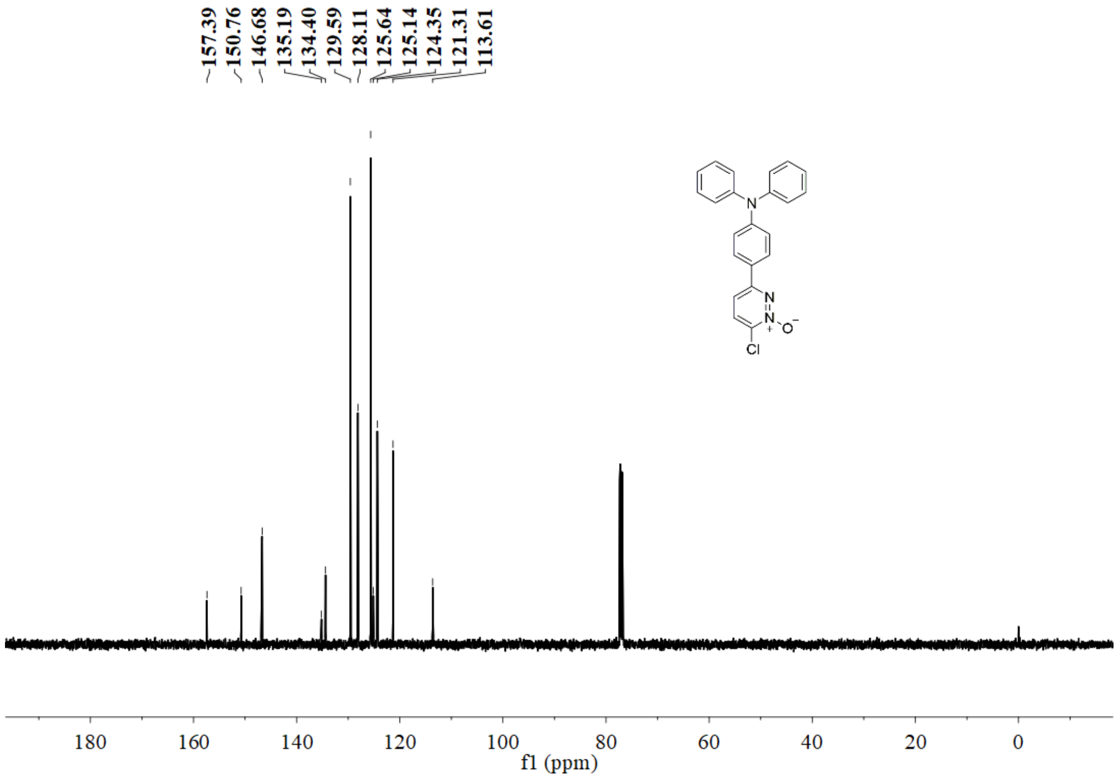

Figure S8. ^{13}C NMR spectrum of **3** in $CDCl_3$.

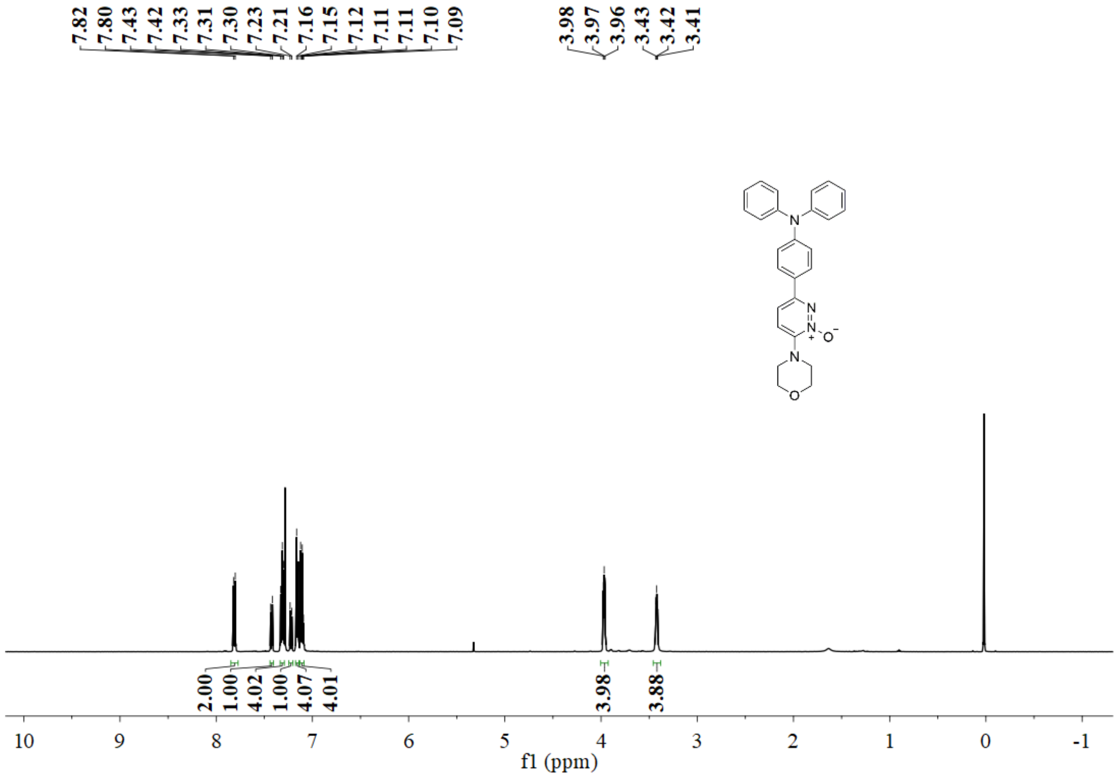

Figure S9. ^{1}H NMR spectrum of MPD-O in $CDCl_3$.

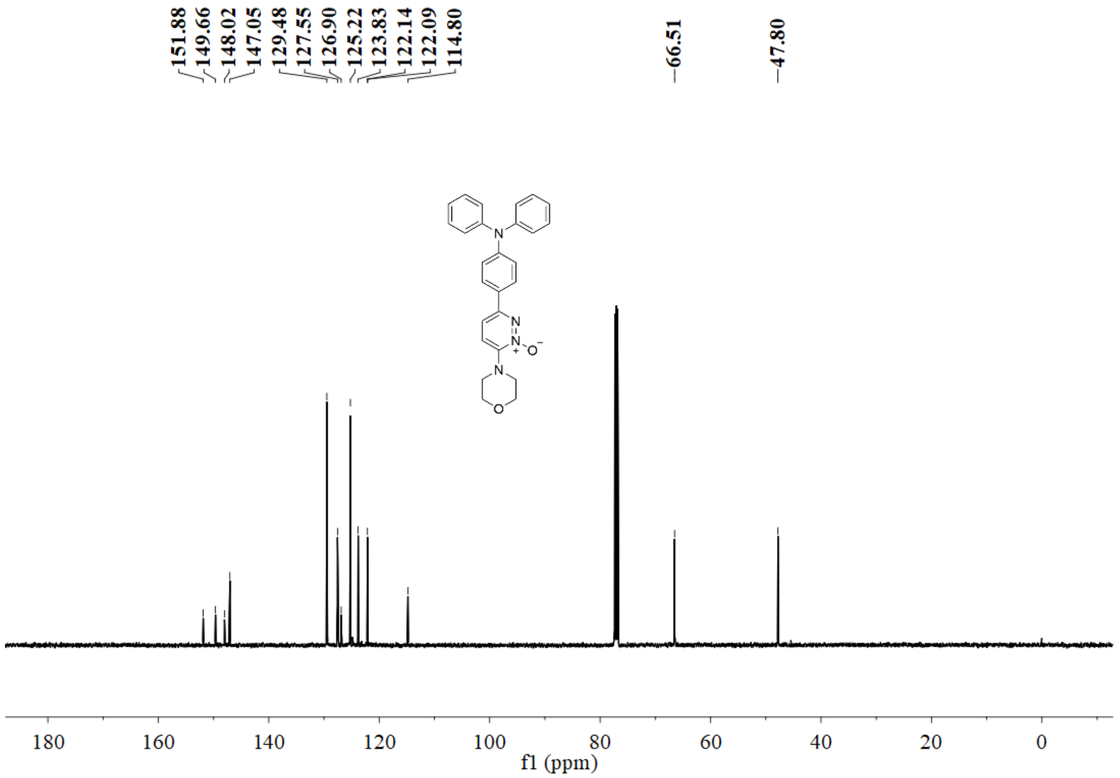

Figure S10. ^{13}C NMR spectrum of MPD-O in $CDCl_3$.

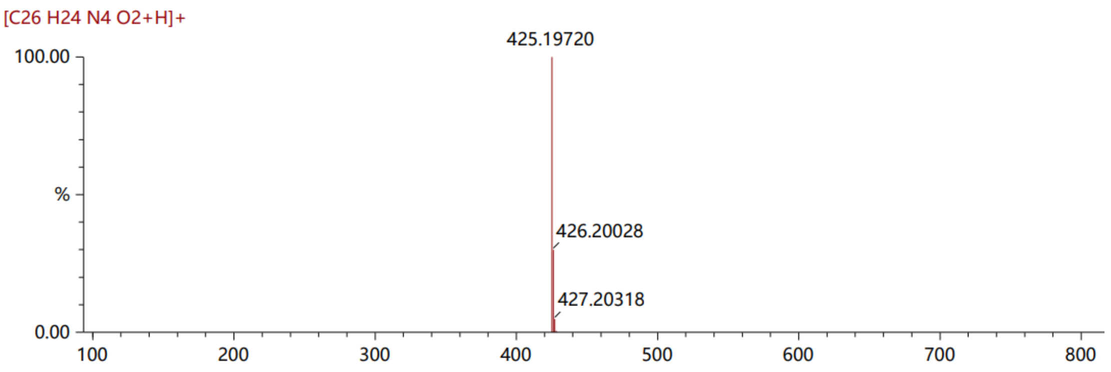

Figure S11. HRMS spectrum of MPD-O.

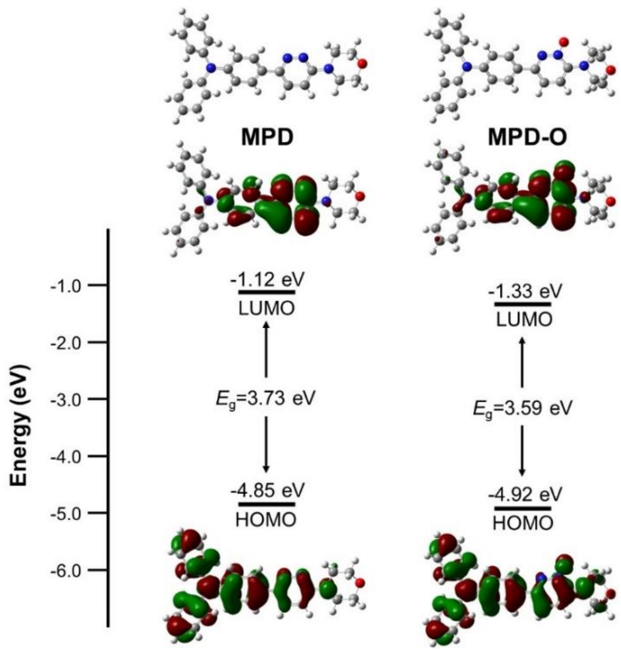

Figure S12. The HOMO and LUMO electron cloud distribution of MPD and MPD-O in gaseous state.

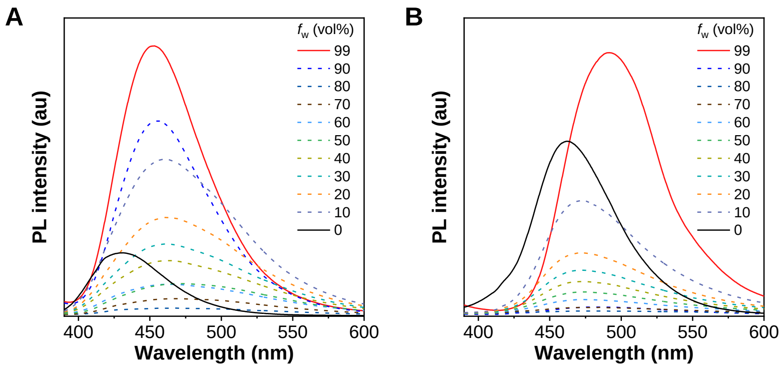

Figure S13. PL spectra of (A) MPD and (B) MPD-O in THF/water mixtures with different water fractions (f_W).

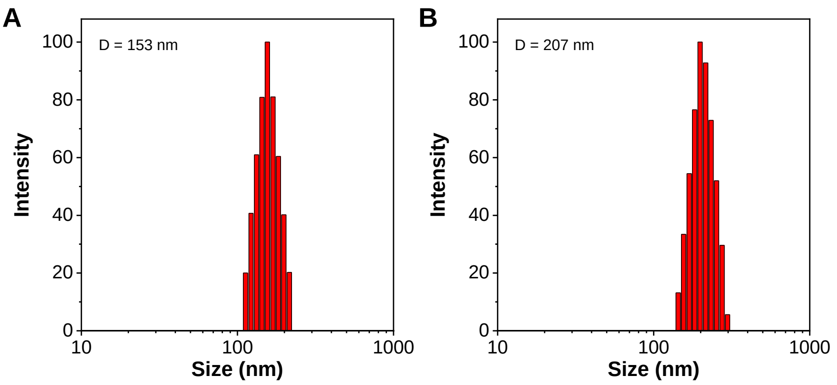

Figure S14. Particle size distribution of (A) MPD and (B) MPD-O in DMSO/H_2O (*v*/*v*, 1/99).

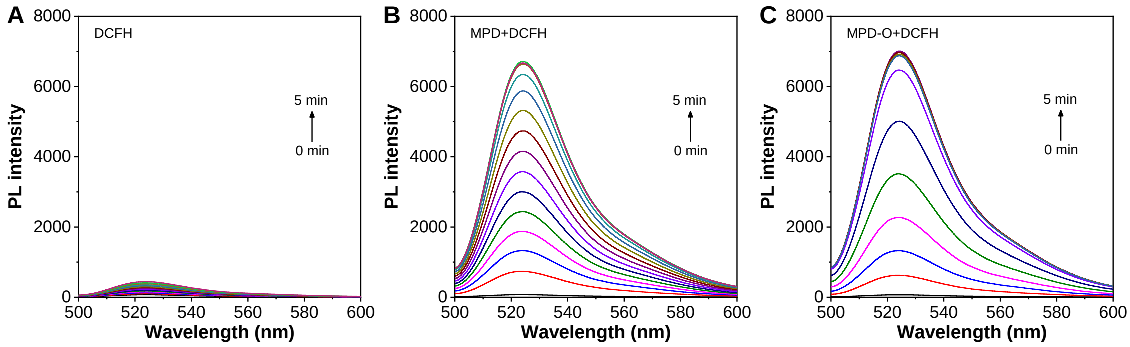

Figure S15. The fluorescence spectra changes of DCFH for ROS detection. (A) DCFH alone, (B) MPD+DCFH and (C) MPD-O+DCFH under white light with different irradiation time. The concentration of MPD or MPD-O is 10 μM; Light power: 100 mW cm^{-2}.

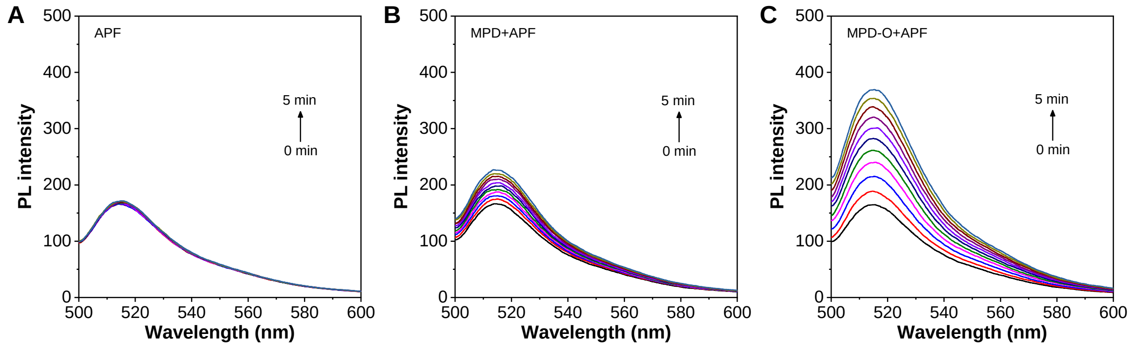

Figure S16. The fluorescence spectra changes of APF. (A) APF alone, (B) MPD+APF and (C) MPD-O+APF under white light with different irradiation time. The concentration of MPD or MPD-O is 10 μM; Light power: 100 mW cm^{-2}.

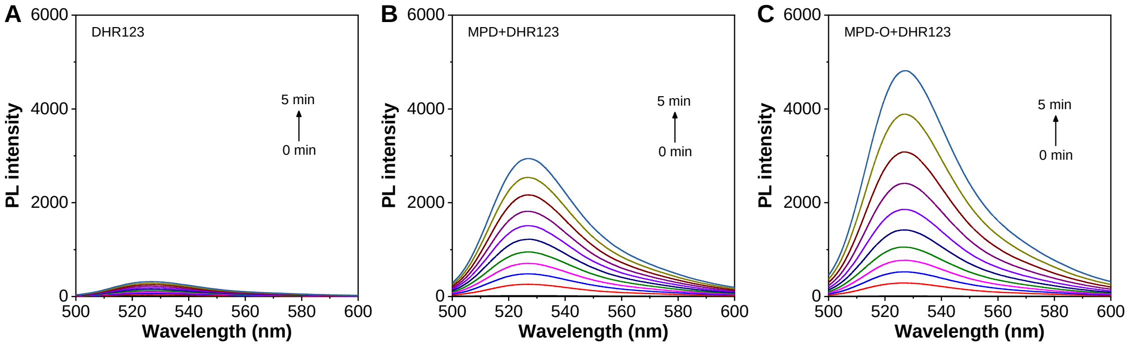

Figure S17. The fluorescence spectra changes of DHR123. (A) DHR123 alone, (B) MPD+DHR123 and (C) MPD-O+DHR123 under white light with different irradiation time. The concentration of MPD or MPD-O is 10 μM; Light power: 100 mW cm^{-2}.

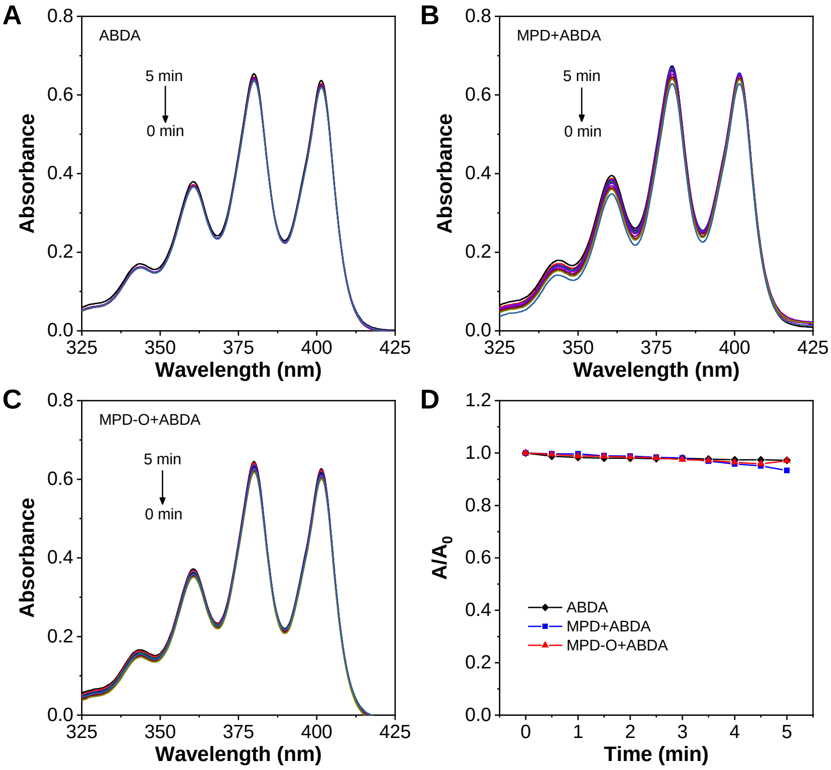

Figure S18. UV-vis spectra changes of ABDA in PBS. (A) ABDA alone, (B) MPD+ABDA and (C) MPD-O+ABDA under white light with different irradiation time. (D) Relative changes in absorbance of ABDA in the presence of MPD and MPD-O upon white light irradiation for different times. The concentration of MPD or MPD-O is 10 μM; Light power: 100 mW cm^{-2}.

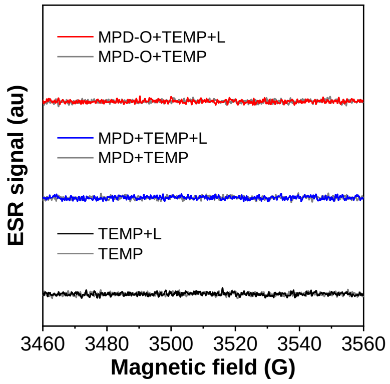

Figure S19. ESR signals of TEMP in the presence/absence of MPD and MPD-O before and after white light irradiation.

Table S1. Crystallographic and structural refinement data of MPD and MPD-O.

Name	MPD	MPD-O
Empirical formula	$C_{26}H_{24}N_4O$	$C_{26}H_{24}N_4O_2$
Formula weight	408.49	424.49
Temperature (K)	100.00(10)	100.0(2)
Wavelength (Å)	0.71073	0.71073
Crystal system	monoclinic	monoclinic
Space group	$P2_1/c$	$P2_1/c$
a (Å)	21.2739(12)	20.8659(13)
b (Å)	11.0135(8)	11.4511(9)
c (Å)	8.6873(5)	8.7949(5)
α (°)	90	90
β (°)	95.211(5)	95.531(6)
γ (°)	90	90
Volume (Å^3)	2027.0(2)	2091.6(2)
Z	4	4
2Θ range for data collection (°)	4.134 to 146.896	4.062 to 49.992
Index ranges	-26 ≤ h ≤ 25, -14 ≤ k ≤ 9, -10 ≤ l ≤ 8	-20 ≤ h ≤ 24, -13 ≤ k ≤ 9, -9 ≤ l ≤ 10
CCDC Number	2210455	2210454

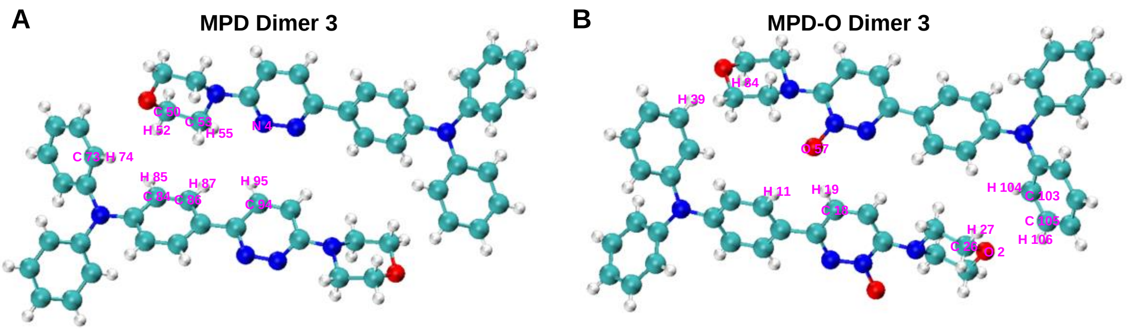

A

Atomic pair	Delta-g index	Percentage contribution
52 73	0.095815	2.03 %
52 84	0.090455	1.92 %
52 74	0.087476	1.85 %
52 85	0.080876	1.71 %
4 95	0.074109	1.57 %
53 86	0.072100	1.53 %
50 74	0.069752	1.48 %
50 73	0.067043	1.42 %
55 87	0.065518	0.39 %
4 94	0.061698	0.31 %

B

Atomic pair	Delta-g index	Percentage contribution
19 57	0.174571	3.76 %
27 103	0.099144	2.14 %
18 57	0.092383	1.99 %
27 105	0.084443	1.82 %
2 106	0.078680	1.70 %
11 57	0.076785	1.66 %
39 84	0.076423	1.65 %
27 104	0.070639	1.52 %
26 103	0.069430	1.50 %
26 105	0.066134	1.43 %

Figure S20. The contribution of ten atomic pairs with the largest percentage to intermolecular interactions of (A) MPD dimer 3 and (B) MPD-O dimer 3.

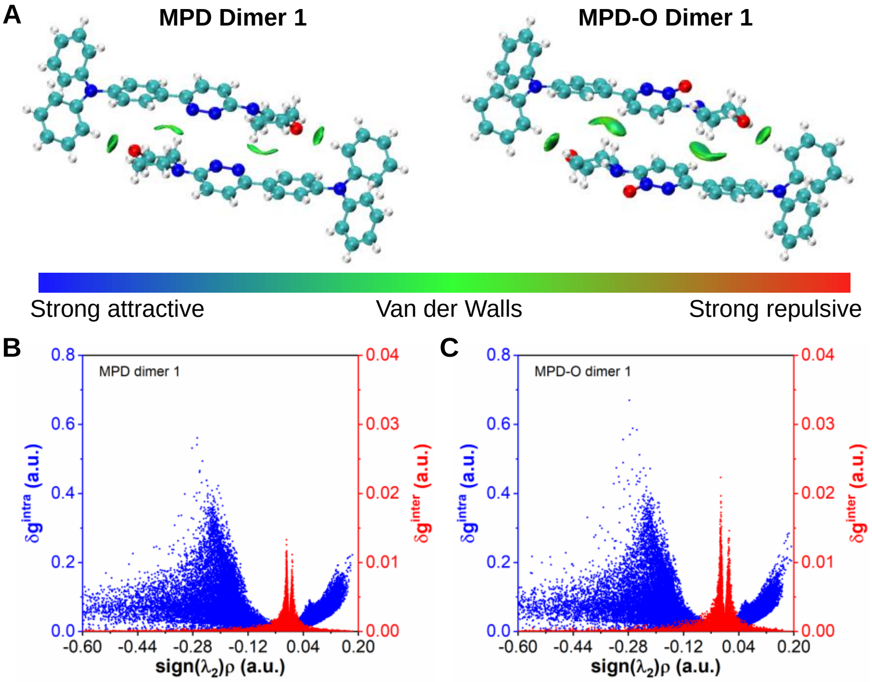

Figure S21. (A) The visualized isosurfaces of the IGM analysis for dimer 1 in MPD or MPD-O ($\delta g^{inter} = 0.007$). The 2D plot of δg^{intra} (blue) and δg^{inter} (red) for (B) MPD dimer 1 and (C) MPD-O dimer 1.

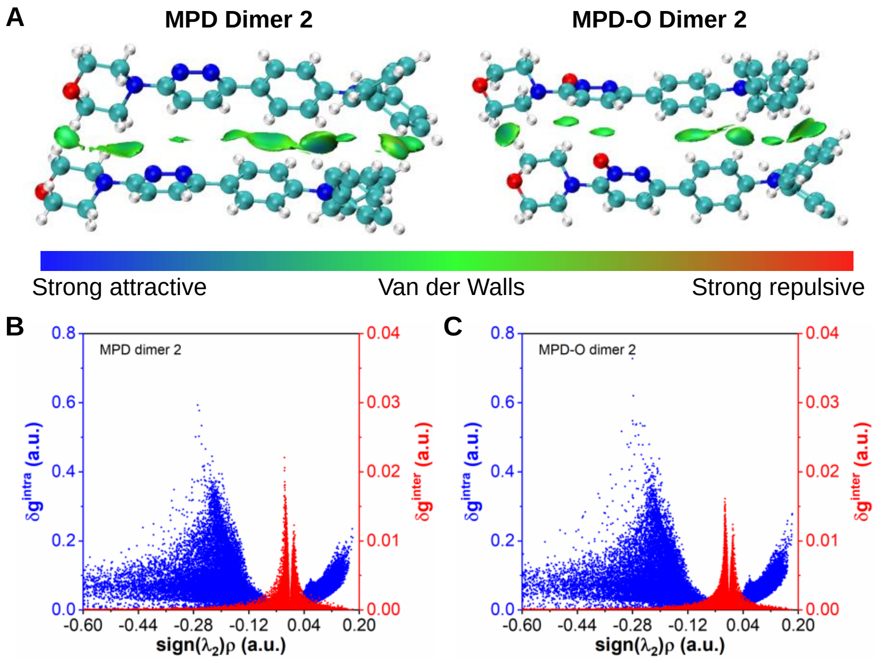

Figure S22. (A) The visualized isosurfaces of the IGM analysis for dimer 2 in MPD or MPD-O ($\delta g^{inter} = 0.007$). The 2D plot of δg^{intra} (blue) and δg^{inter} (red) for (B) MPD dimer 2 and (C) MPD-O dimer 2.

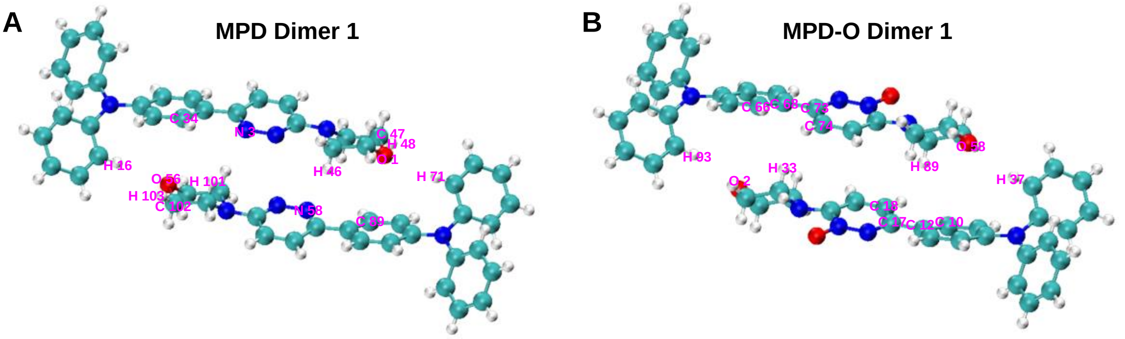

Atomic pair	Delta-g index	Percentage contribution
16 56	0.077913	1.62 %
1 71	0.077913	1.62 %
46 58	0.068914	1.43 %
3 101	0.068853	1.43 %
16 103	0.062722	1.30 %
48 71	0.062672	1.30 %
16 102	0.061666	1.28 %
47 71	0.061663	1.28 %
46 89	0.057946	1.20 %
34 101	0.057896	1.20 %

Atomic pair	Delta-g index	Percentage contribution
2 93	0.106194	1.79 %
37 58	0.106174	1.79 %
18 89	0.098506	1.66 %
33 74	0.098500	1.66 %
12 89	0.078666	1.32 %
33 68	0.078666	1.32 %
33 73	0.076045	1.28 %
17 89	0.076000	1.28 %
10 89	0.074872	1.26 %
33 66	0.074785	1.26 %

Figure S23. The contribution of ten atomic pairs with the largest percentage to intermolecular interactions of (A) MPD dimer 1 and (B) MPD-O dimer 1.

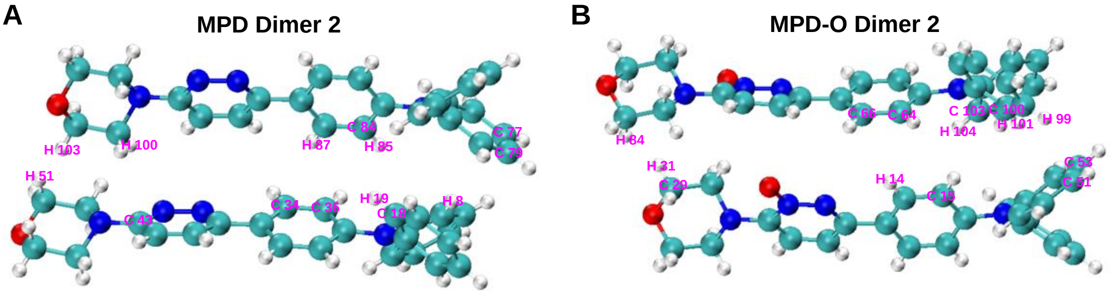

(A) MPD Dimer 2

Atomic pair	Delta-g index	Percentage contribution
19 84	0.117715	1.43 %
36 87	0.096244	1.17 %
8 79	0.091099	1.11 %
43 100	0.090527	1.10 %
18 84	0.087807	1.07 %
34 87	0.086342	1.05 %
8 77	0.084850	1.03 %
19 85	0.082558	1.01 %
51 103	0.080107	0.98 %
18 85	0.077633	0.95 %

(B) MPD-O Dimer 2

Atomic pair	Delta-g index	Percentage contribution
15 104	0.096823	1.35 %
53 101	0.088516	1.23 %
31 84	0.087250	1.21 %
51 101	0.084878	1.18 %
14 64	0.078111	1.09 %
51 99	0.075839	1.05 %
14 66	0.072185	1.00 %
15 103	0.071301	0.99 %
29 84	0.069404	0.96 %
51 100	0.068621	0.95 %

Figure S24. The contribution of ten atomic pairs with the largest percentage to intermolecular interactions of (A) MPD dimer 2 and (B) MPD-O dimer 2.

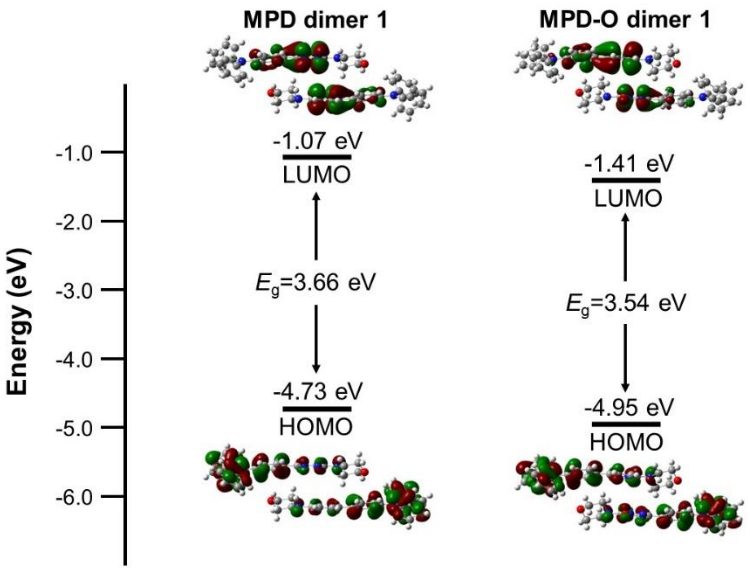

Figure S25. The HOMO and LUMO electron cloud distribution of MPD dimer 1 and MPD-O dimer 1.

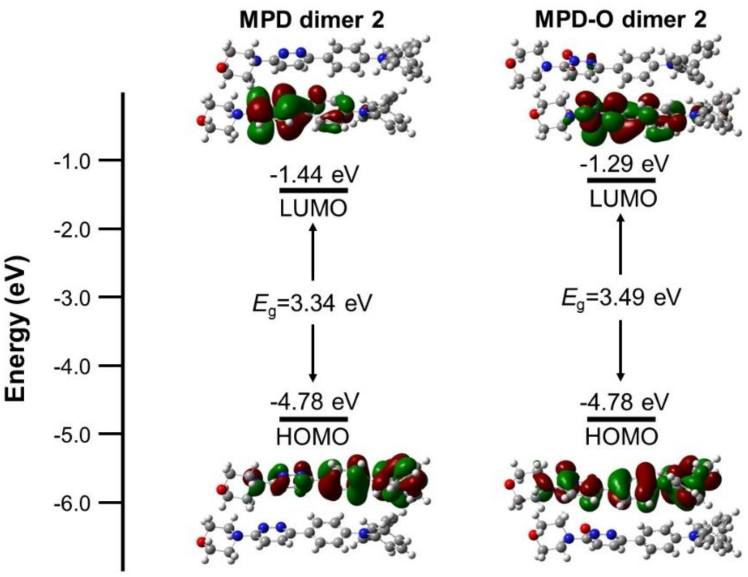

Figure S26. The HOMO and LUMO electron cloud distribution of MPD dimer 2 and MPD-O dimer 2.

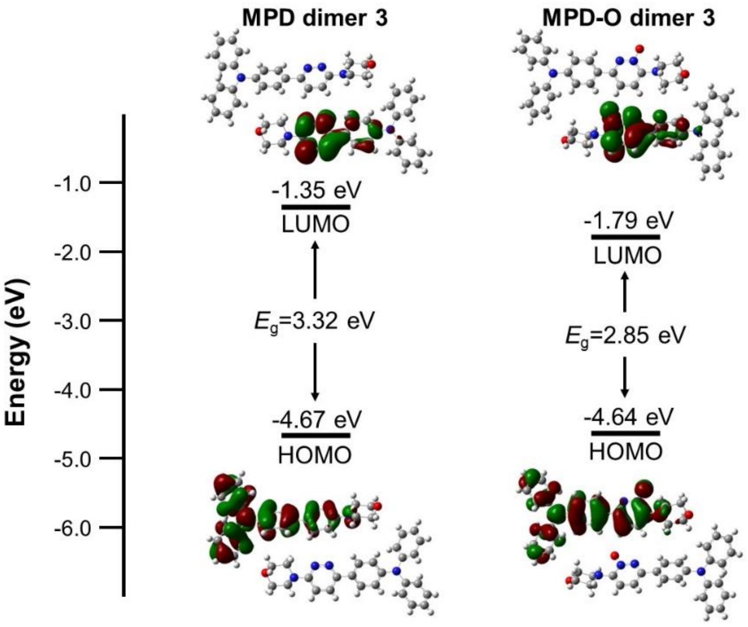

Figure S27. The HOMO and LUMO electron cloud distribution of MPD dimer 3 and MPD-O dimer 3.

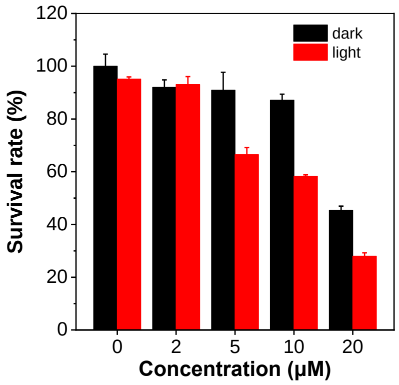

Figure S28. Bacteria survival rates of *S. aureus* incubated with the various concentrations of MPD-O in darkness or upon white light irradiation (100 mW cm^{-2}).

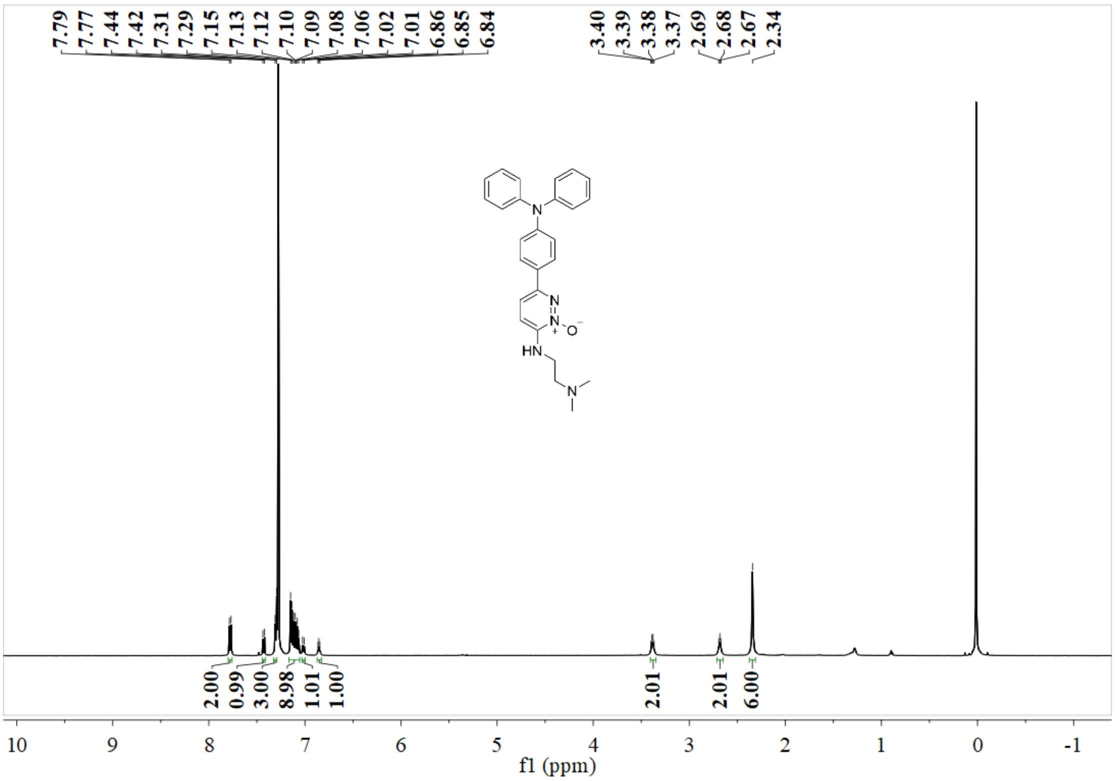

Figure S29. ^{1}H NMR spectrum of **4** in $CDCl_3$.

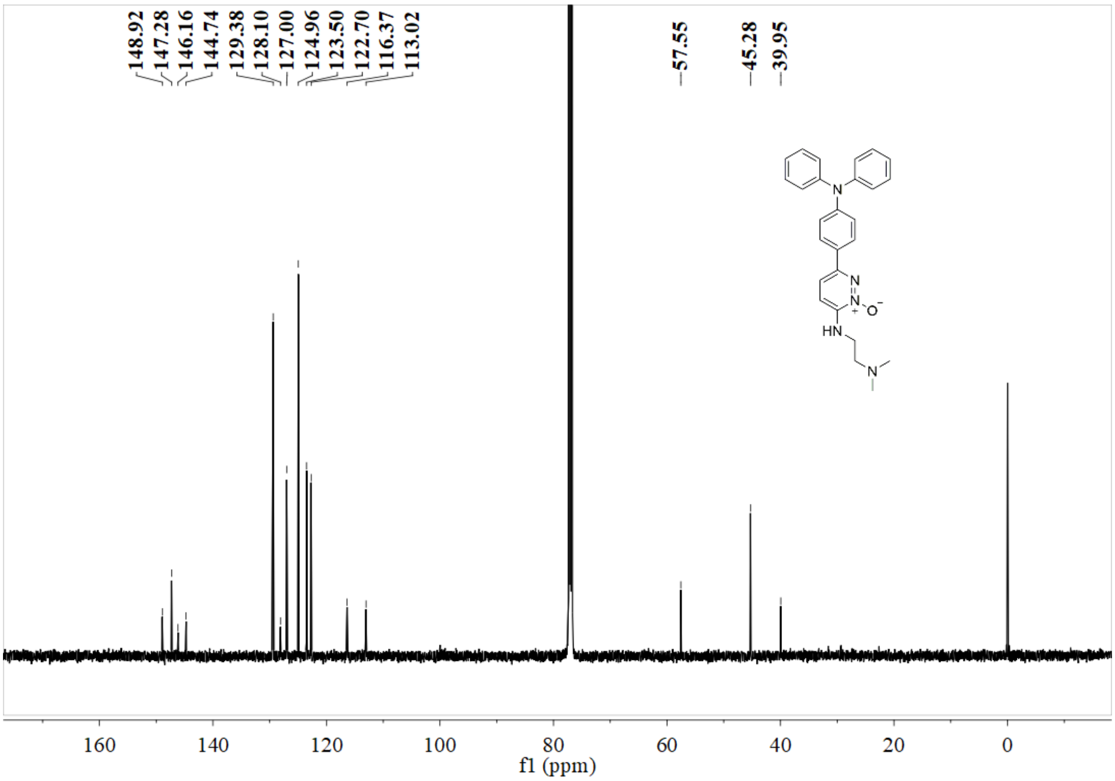

Figure S30. ^{13}C NMR spectrum of **4** in $CDCl_3$.

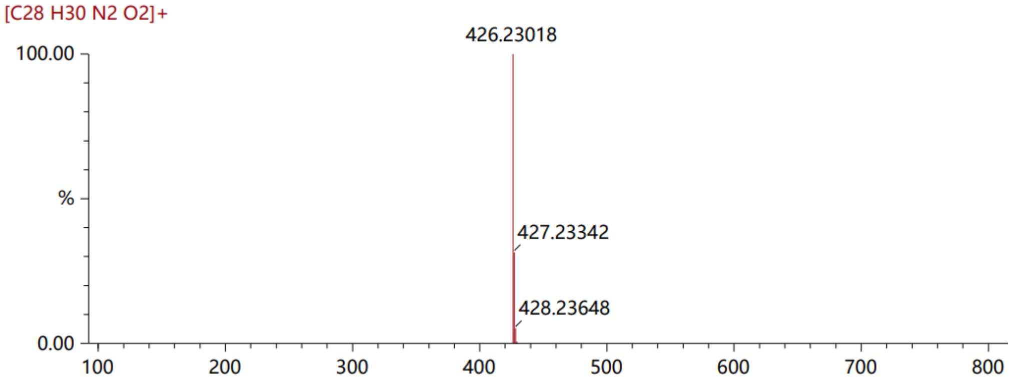

Figure S31. HRMS spectrum of **4**.

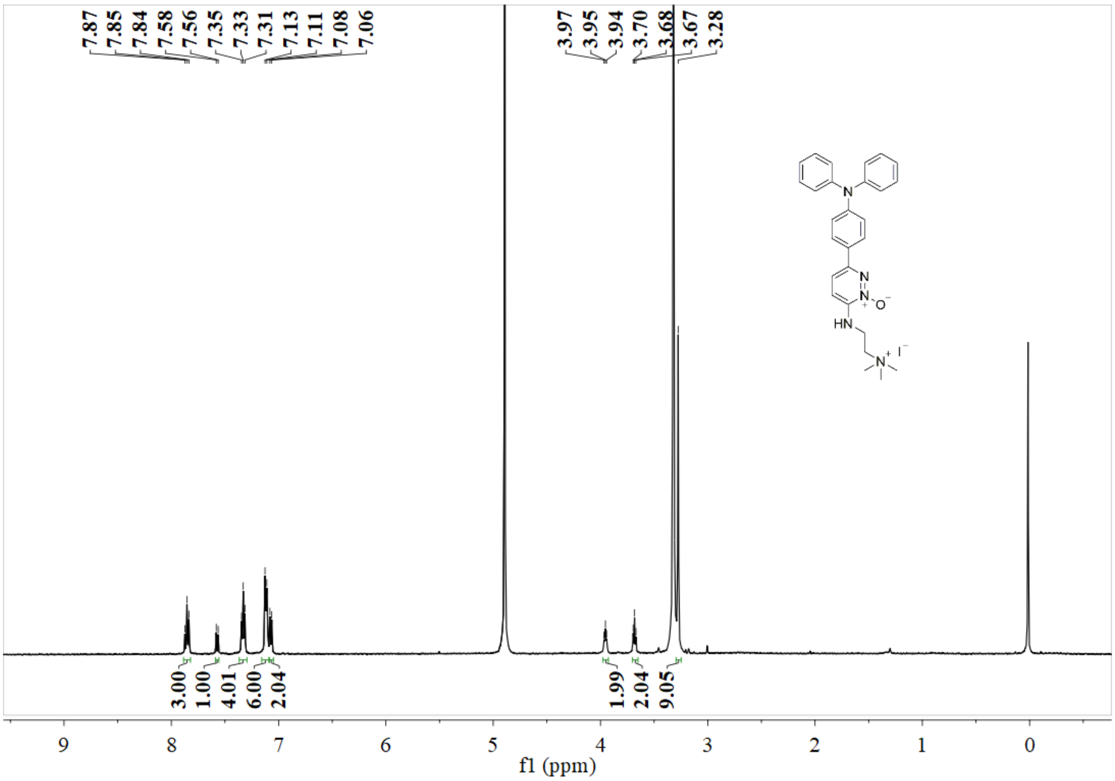

Figure S32. ^{1}H NMR spectrum of DAPD-O in CD_3OD.

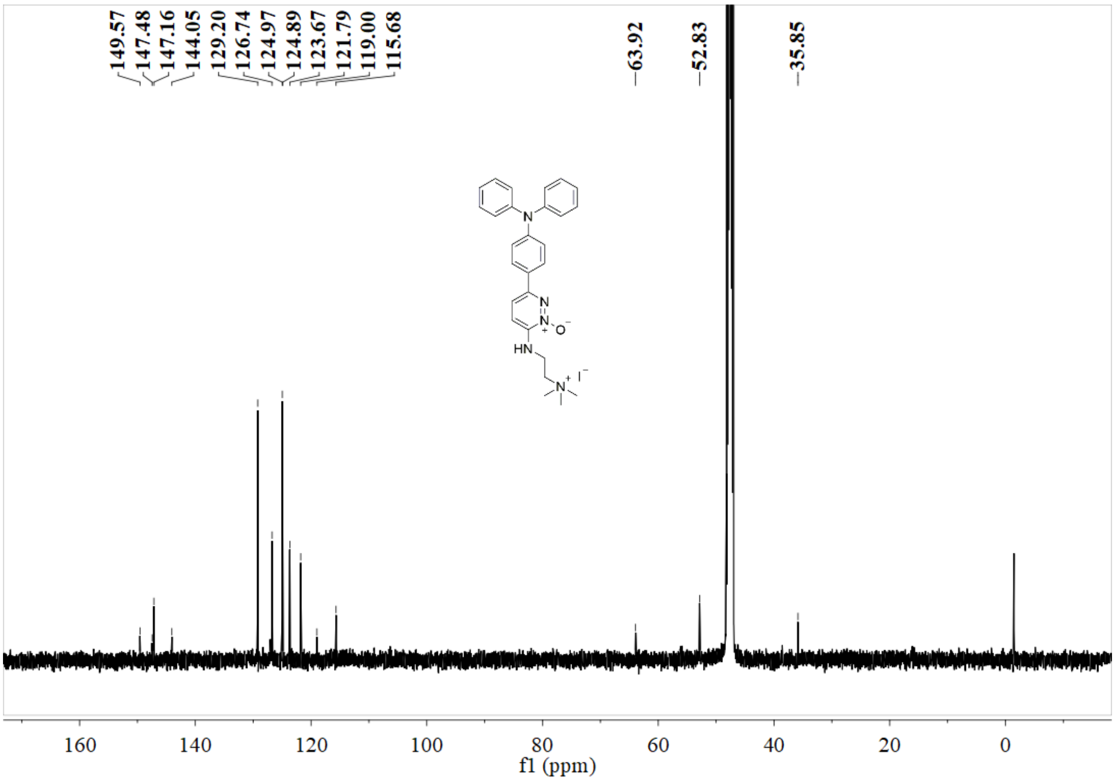

Figure S33. ^{13}C NMR spectrum of DAPD-O in CD_3OD.

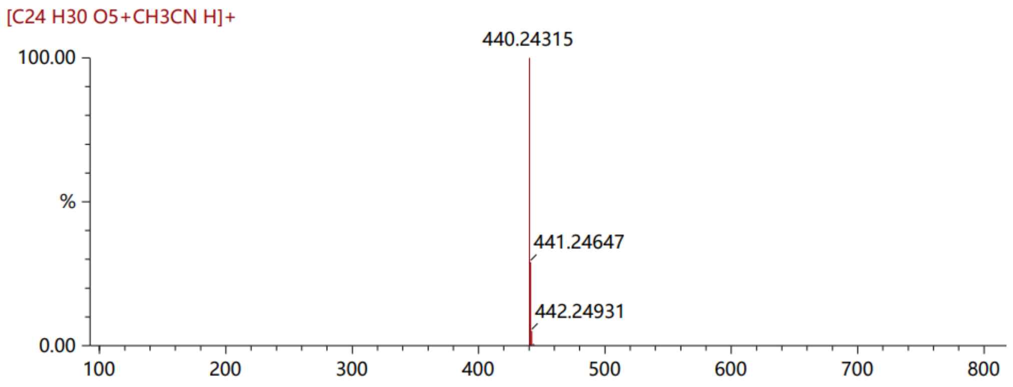

Figure S34. HRMS spectrum of DAPD-O.

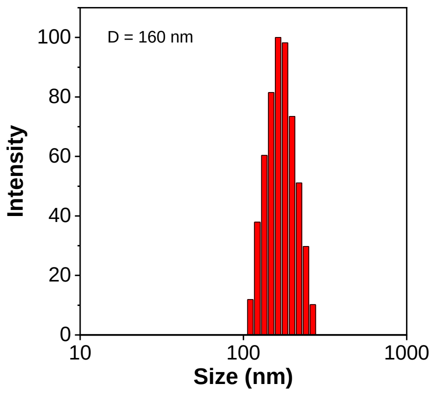

Figure S35. Particle size distribution of DAPD-O in DMSO/H_2O (*v*/*v*, 1/99).

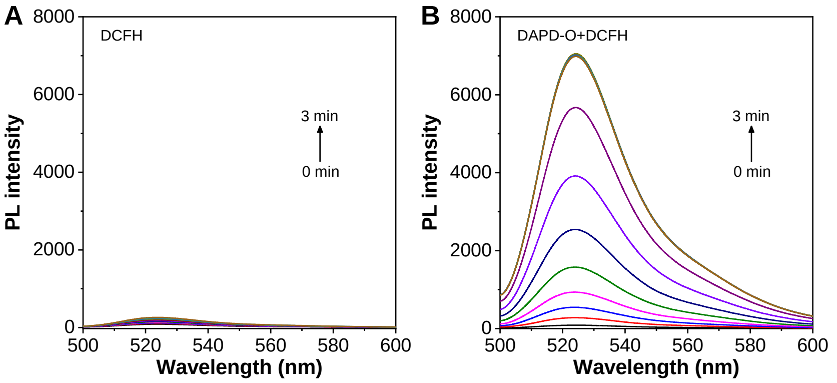

Figure S36. The fluorescence spectra changes of DCFH. (A) DCFH alone, (B) DAPD-O+DCFH under white light with different irradiation time. The concentration of DAPD-O is 10 μM; Light power: 100 mW cm^{-2}.

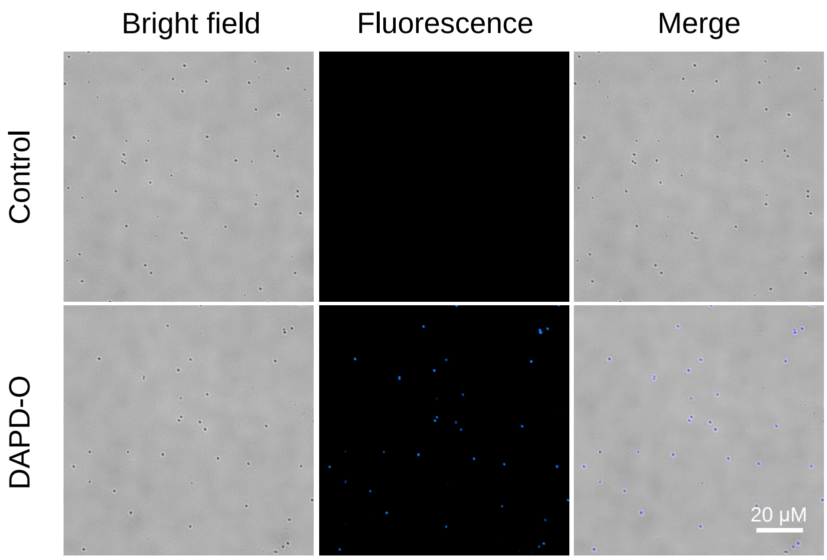

Figure S37. Bright field and fluorescent images of *S. aureus* incubated with DAPD-O (10 μM) for 2 h.

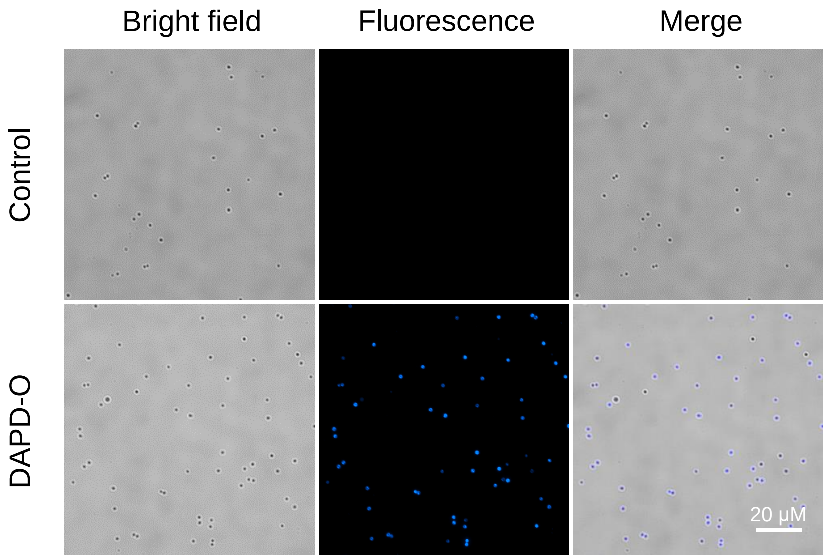

Figure S38. Bright field and fluorescent images of *MRSA* incubated with DAPD-O (10 μM) for 2 h.

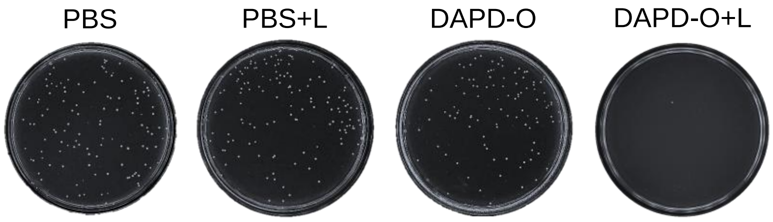

Figure S39. Photographs of *S. aureus* cultured on agar plate after being treated with PBS or DAPD-O (10 μM).

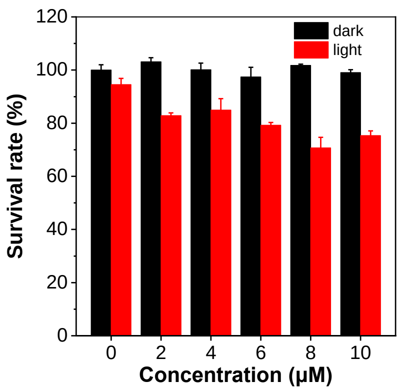

Figure S40. Bacteria survival rates of *E.coli* incubated with the various concentrations of DAPD-O in darkness or upon white light irradiation (100 mW cm^{-2}).

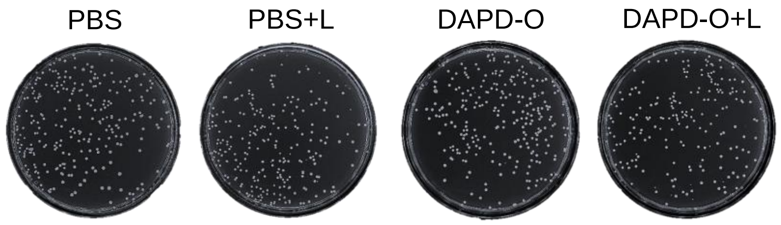

Figure S41. Photographs of *E.coli* cultured on agar plate after being treated with PBS or DAPD-O (10 μM).

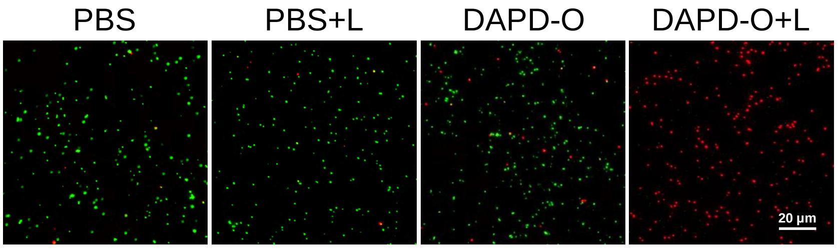

Figure S42. Live/dead bacteria staining images of *S. aureus* after being treated with PBS or DAPD-O (10 μM) with/without white light irradiation. Green fluorescence represents surviving bacteria, and red fluorescence represents dead bacteria.

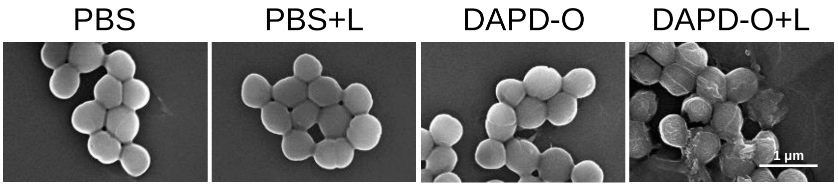

Figure S43. SEM images of *S. aureus* after being treated with PBS or DAPD-O (10 μM) with/without white light irradiation.

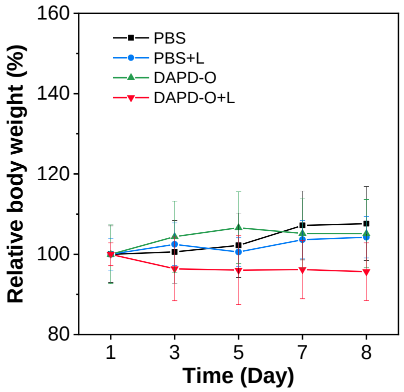

Figure S44. Body weight curve of *MRSA* infected mice during the wound healing process after different treatments.

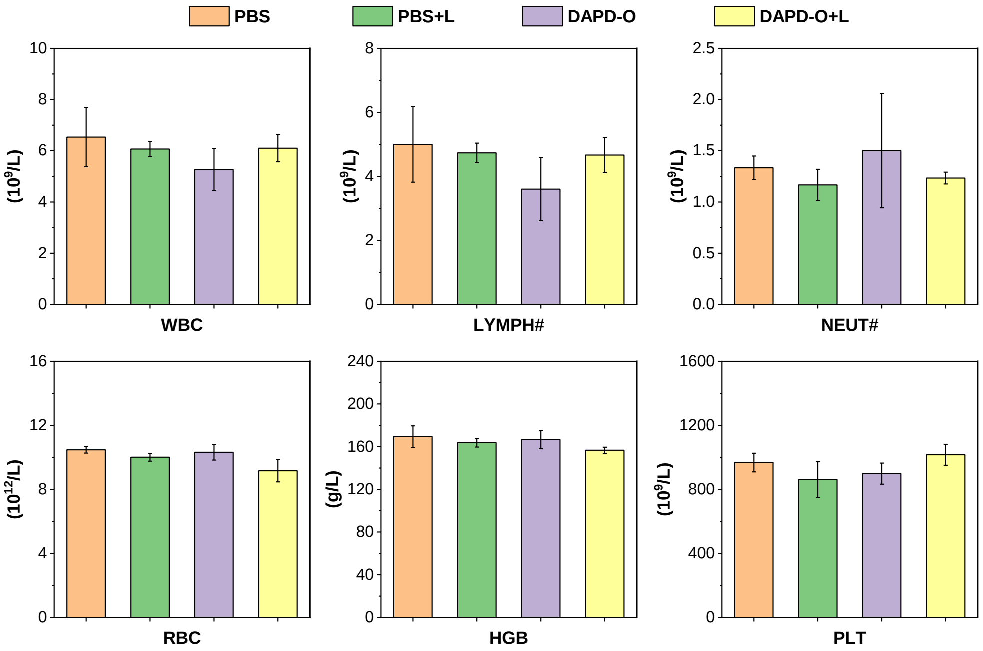

Figure S45. Blood routine assays of *MRSA* infected mice at day 8 after different treatments.

Supplemental References

(1) Gao, X.; Bai, S.; Fazzi, D.; Niehaus, T.; Barbatti, M.; Thiel, W. Evaluation of Spin-Orbit Couplings with Linear-Response Time-Dependent Density Functional Methods. *J. Chem. Theory Comput.* **2017**, *13*, 515-524.

(2) Chiodo, S. G.; Leopoldini, M. MolSOC: A Spin–Orbit Coupling Code. *Comput. Phys. Commun.* **2014**, *185*, 676-683.

(3) Lefebvre, C.; Rubez, G.; Khartabil, H.; Boisson, J. C.; Contreras-Garcia, J.; Henon, E. Accurately Extracting the Signature of Intermolecular Interactions Present in the NCI Plot of the Reduced Density Gradient versus Electron Density. *Phys. Chem. Chem. Phys.* **2017**, *19*, 17928-17936.

(4) Lu, T.; Chen, Q. Independent Gradient Model Based on Hirshfeld Partition: A New Method for Visual Study of Interactions in Chemical Systems. *J. Comput. Chem.* **2022**, *43*, 539-555.

(5) Lu, T.; Chen, F. Multiwfn: A Multifunctional Wavefunction Analyzer. *J. Comput. Chem.* **2012**, *33*, 580-592.

(6) Humphrey, W.; Dalke, A.; Schulten, K. VMD: Visual Molecular Dynamics. *J. Mol. Graphics.* **1996**, *14*, 33-38.
